# Controllable assembly of synthetic constructs with programmable ternary DNA interaction

**DOI:** 10.1093/nar/gkac478

**Published:** 2022-06-17

**Authors:** Huangchen Cui, Tianqing Zhang, Yuhan Kong, Hang Xing, Bryan Wei

**Affiliations:** School of Life Sciences, Tsinghua University-Peking University Center for Life Sciences, Center for Synthetic and Systems Biology, Tsinghua University, Beijing 100084, China; School of Life Sciences, Tsinghua University-Peking University Center for Life Sciences, Center for Synthetic and Systems Biology, Tsinghua University, Beijing 100084, China; Institute of Chemical Biology and Nanomedicine; State Key Laboratory of Chemo/Biosensing and Chemometrics; Hunan Provincial Key Laboratory of Biomacromolecular Chemical Biology; College of Chemistry and Chemical Engineering; Hunan University, Changsha 410082, China; Institute of Chemical Biology and Nanomedicine; State Key Laboratory of Chemo/Biosensing and Chemometrics; Hunan Provincial Key Laboratory of Biomacromolecular Chemical Biology; College of Chemistry and Chemical Engineering; Hunan University, Changsha 410082, China; School of Life Sciences, Tsinghua University-Peking University Center for Life Sciences, Center for Synthetic and Systems Biology, Tsinghua University, Beijing 100084, China

## Abstract

Compared with the dual binding components in a binary interaction, the third component of a ternary interaction often serves as modulator or regulator in biochemical processes. Here, we presented a programmable ternary interaction strategy based on the natural DNA triplex structure. With the DNA triplex-based ternary interaction, we have successfully demonstrated controllable hierarchical assemblies from nanometer scale synthetic DNA nanostructure units to micrometer scale live bacteria. A selective signaling system responsive to orthogonal nucleic acid signals via ternary interaction was also demonstrated. This assembly method could further enrich the diversified design schemes of DNA nanotechnology.

## INTRODUCTION

In the past few decades, tremendous efforts have been devoted for the grand goal of creating materials of novel features by the programmable arrangements of individual building blocks (1–4). Inspired by versatile biomacromolecules (5–8) that are modulated and controlled from a myriad of interacting components, such as nucleobases, amino acids, and phospholipids, stimulus-responsive assembly (9–11) becomes an attractive strategy to achieve this goal. However, the goal of creating synthetic systems with stimuli-responsive properties to mimic natural macromolecules remains challenging. Notably, utilizing ternary interaction with the third component as a programmable modulator for regulating binding activities on the interface stands out as a promising framework to implement dynamic assembly responsive to specific signals.

DNA nanostructures and especially constructs from DNA origami approach with high programmability and complexity have emerged as an excellent smart material for scientific investigations in responsive assembly (12–17). Recent progress of fabricating nanofibrils with DNA origami units as Janus particles also highlights the adaptability of DNA origami to investigate diverse binary interactions (18–23) (e.g. DNA–DNA interactions, coiled-coil interactions, and adamantane-βCyclodextrin interactions).

**Figure 1.**
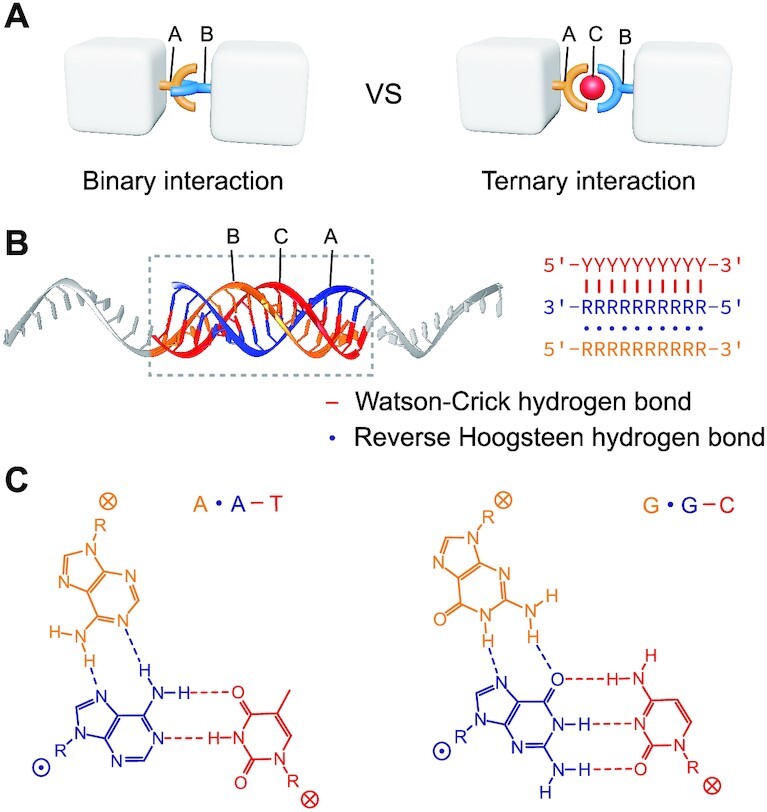
Ternary interaction based on DNA triplex. (**A**) Model schematics of binary interaction versus ternary interaction. In binary interaction, the two moieties A and B (blue and yellow) directly bind together once they encounter; in ternary interaction, the binding relies on the component C (red) which functions as a modulator. (**B**) Model schematics (left) and strand diagram (right) of antiparallel DNA triplex as ternary interaction. Nucleotide base codes: Y (T and C), R (A and G). Watson–Crick and reverse Hoogsteen interactions were depicted as (−) and (⋅), respectively. (**C**) Nucleotide base triplets of A⋅A–T and G⋅G–C.

Here, we sought to apply DNA triplex-based ternary interaction (A–B–C) on simple synthetic DNA tile structures as well as Janus DNA origami cuboids to study the corresponding responsive assembly upon the addition of third component. Unlike the common binary interaction, our DNA triplex ternary interaction comprise three strands: A, B and C, where C serves as an external signal (Figure 1A) to induce indirect interaction between A and B.

Based on the investigations on simple DNA tiles, we applied DNA triplex interaction in a Janus DNA origami cuboid, in which the strands A and B of this ternary interaction were appended to origami cuboids. Origami cuboids appended with strands A and B were designed to stay as dispersed particles (inactive) initially, and in turn, assembled into nanofibrils (active) via A–B–C ternary interaction triggered by the modulator (strand C). In our implementation of responsive assembly of dimers, trimers, and oligomers of origami cuboids, morphological and statistical analysis demonstrated the high controllability of this design strategy. This ternary interaction was further applied to a selective signaling system where the assembly process of origami units can be orthogonally controlled by external signals. Moreover, we successfully applied the ternary interaction strategy to regulate the organization of bacterial cells, extending this type of regulatory assembly to not only synthetic biomolecules of nanometer scale but also living microorganisms of micrometer scale.

## MATERIALS AND METHODS

### Reagents

M13mp18 DNA scaffold was purchased from Bioruler Co. Ltd (China). All short oligonucleotides were purchased from Integrated DNA Technology Inc. (USA) and Sangon Biotech Co. Ltd (China). Reagent-grade chemicals were commercially available and were used without further purifications.

### Preparation of DNA duplex and double-crossover (DX) motifs

DNA duplex sequences were generated by NUPACK (24). DX motifs were adapted from the design of Stephanopoulos and coworkers (21). DNA strands with equal molar ratio were mixed in 1× **Buffer 1** solution [40 mM tris base, 20 mM acetic acid, 1 mM EDTA and 12.5 mM magnesium chloride, pH ∼ 8;] at final concentration of 1 μM. 50 μl solution was annealed: 95°C for 5 min followed by a gradient from 90 to 40°C by −1°C/2 min and hold at 35°C for 1–2 h in a Nexus X2 Mastercycler (Eppendorf).

### Preparation of monomeric DNA origami cuboids

DNA origami cuboids were adapted from the design of Walther and coworkers (20). For all DNA origami cuboids, final concentration of M13mp18 strand in 1 × **Buffer 2** solution [40 mM tris base, 20 mM acetic acid, 1 mM EDTA and 20 mM magnesium chloride, pH ∼ 8;] was 10 nM and final concentration of each DNA staple strand (including core staple strands and edge staple strands) was 50 nM. For origami with valency number of N, additional anchor strands (A* strand to bind with cuboid left handles, and B* strand to bind with cuboid right handles) were added at the stoichiometric equivalent to handle strands. Solution was annealed using the following protocol: 65°C for 15 min followed by a gradient from 60 to 40°C by −0.5°C/30 min.

### Dimerization and trimerization of DNA origami cuboids

Origami ‘Cuboid_I_’ and ‘Cuboid_II_’ were prepared and annealed separately and mixed with strand C at the stoichiometric equivalent amount (1:1:9 for dimerization and 1:2:18 or 2:1:18 for trimerization) and incubated at 35°C overnight.

### One-pot polymerization of DNA origami cuboids

Samples were prepared with the same method as monomerization except for adding stoichiometric equivalent amount of the strand C in sample solution, then annealed with the following protocol: 65°C for 15 min followed by a gradient from 60 to 40°C by −0.5°C/30 min and hold at 35°C overnight.

### Native agarose gel electrophoresis (AGE)

4% native agarose gel (for duplex structures and DX tiles) or 1% native agarose gel (for DNA origami cuboids) was prepared with 0.5× TBE (10 mM Mg^2+^) as the running buffer. Gels were run at 90 V (constant voltage) for 60–120 min. After electrophoresis, gel was stained by 1 × SYBR Safe (Thermo Fisher Scientific Inc.) and scanned in Amersham Typhoon Scanner (GE Healthcare) at designative channel.

### Transmission electron microscopy (TEM) imaging

Carbon-coated copper grids (Ted Pella Inc. or Beijing XXBR Technology Co. Ltd) were plasma cleaned for 30 s. 5–10 μl sample was loaded onto the grid and incubated for 2 min. The solution was removed from the grid with filter paper. Then 5 μl of 2% aqueous uranyl acetate solution was added to the grid and incubated for 15 s. After the uranyl acetate solution was removed, copper grid was allowed to dry for ∼2 min. Samples were imaged via a FEI Tecnai Spirit transmission electron microscope (120 kV). TEM images were quantified using software ImageJ.

### Structure illumination microscopy (SIM) sample preparation

A tape microfluidic was prepared by first cleaning a microscope slide and glass coverslip (#1.5 22 × 22 mm^2^, VWR) in an air plasma cleaner for 30 s. Next, thin strips of double-sided tape (Scotch Permanent) were adhered to the microscope slide in parallel to create channels ∼2 mm in diameter. Coverslip was placed on top of the double-sided tape and firmly adhered by applying gentle pressure. To facilitate the charge-mediated adsorption of the origami cuboids to the plasma-cleaned glass, unpurified samples were first diluted to the working concentration of 100 pM with buffer (0.5 × TBE,10 mM MgCl_2_, ∼ pH 9.1). Diluted samples were added to the tape microfluidic. Images were taken on a Nikon Eclipse Ti microscope, operated by Nikon Elements software.

### SIM results analyses

The data in raw .nd format was converted into .tiff format and analyzed with ImageJ. Briefly, images were converted to 8-bit pure grayscale and set with a proper threshold for quantification, then analyzed through ‘Analyze-Set Measurements’ – ‘Area’ – ‘Bouning rectangle’ – ‘Mean gray value’ – ‘Area fraction’ – ‘Perimeter’ – ‘Limit to threshold’. Next, ‘Analyze-Analyze particles’ option was used to automatically pick all fluorescent dots. Fluorescent dots with intensity higher than 10 were counted and those with intensity higher than 50 were record as origami nanofibrils. Data were collected and yields of origami nanofibrils were calculated.

### Metabolic labeling of bacteria

*E. coli* GFP and RFP were cultivated overnight in liquid LB culture medium supplemented with 25 mg/ml chloramphenicol. Bacterial solution was inoculated into LB liquid medium containing 25 mg/mL chloramphenicol and azido glucose at 1:1000 volume ratio and cultivated for 20 h at 37°C.

### Flow cytometry and confocal characterization of DNA-labelled bacteria

After metabolic labeling, 1 ml of *E. coli* RFP solution was centrifuged at 3000 × g for 3 min and washed with PBS buffer for three times, and resuspended in PBS buffer. Then, 1 μl DBCO-DNA-FAM was co-incubated with bacterial PBS solution at 25°C for 4 h to enable click reaction under vigorous shake. DNA-labelled *E. coli* RFP was collected and washed for three times and resuspended in PBS buffer for subsequent flow cytometry experiments. Bacterial cells were analyzed in a BD Biosciences Accuri C6 flow cytometer by counting 10,000 objects. FAM DNA-labelled *E. coli* RFP solution of 5 μl was added onto slide for observation using confocal laser scanning microscope (Nikon Ti-E + A1RMP + N-STORM).

### DNA duplex-modified bacteria

Functionalized DNA duplexes AA* and BB* were prepared with equal molar ratio and annealed in 0.9% NaCl supplemented with 20 mM Mg^2+^ Buffer. Strand A (or B) possesses an DBCO group at its 5' terminal for bacterial surface ligation and a sticky end for hybridization with strand A* (or B*) whose sequence contains polypurine part for triplex formation. Strands A* and B* were designed with different lengths and named as: A10*, A12*, A16*, A20*, A24* and B10*, B12*, B16*, B20*, B24*. Click reaction between DNA duplex and corresponding bacteria cells was performed in PBS buffer at 25°C for 4 h, after which the AA* modified *E. coli* GFP and BB* modified *E. coli* RFP were collected, respectively.

### DNA ternary interaction mediated bacterial assembly

AA* modified *E. coli* GFP and BB* modified *E. coli* RFP were resuspended in 0.9% NaCl (supplemented with 20 mM Mg^2+^). OD_600_ was adjusted to 0.5 to prevent undesired nonspecific clustering. 10 μl strand C (100 μM) was added into mixed bacterial solution and incubated for 5 h at 37°C. 10 μl clustered bacteria solution was dropped onto the slide and left for 5 min and then covered by a cover glass for observation. Images were acquired on an inverted confocal laser scanning microscope (Nikon Ti-E + A1RMP + N-STORM) equipped with a 488 and 561 nm laser for imaging GFP and RFP, respectively, using a 40× objective lens.

## RESULTS

In this study, we adopted the antiparallel DNA triplex (25–29) as the model of ternary interaction. The antiparallel DNA triplex consists of three DNA strands, one polypyrimidine strand (strand C, red) and two polypurine strands (strands A and B, blue and yellow respectively), in which A⋅A–T and G⋅G–C triplets are presented (Figure 1B). Different from the parallel DNA triplex (Hoogsteen base pairing) commonly used in pH-controlled processes (30,31), the integrity of antiparallel triplex structure is maintained by Watson-Crick base pairing (−) between the polypyrimidine strand and its complementary polypurine strand, and reverse Hoogsteen base pairing (⋅) between two polypurine strands (Figure 1 B-C). Notably, the antiparallel triplex structure is pH-independent and compatible in a wide range of buffer conditions (including physiological conditions), which is not the case for parallel triplex due to its reliance on protonated cytosine (C^+^). In addition, a T⋅A–T triplet is also achievable by a GT-rich strand with complementary polypurine-polypyrimidine duplex, but the binding pattern (parallel or antiparallel) of the GT-rich strand in triplex formation is not well-controlled (29,32). Because of the ambiguity, such a GT-motif triplex model was not considered in this study.

According to the binding test of DNA triplexes whose lengths (L, base numbers of each strand) range from 6 base triplets (bt) to 14 bt, we found that 10-bt triplex demonstrated the optimal binding performance (native agarose gel electrophoresis (AGE) analysis in Supplementary Figure S1). Notably, the ternary interaction remained stable as high as 40 °C (Supplementary Figure S2).

**Figure 2.**
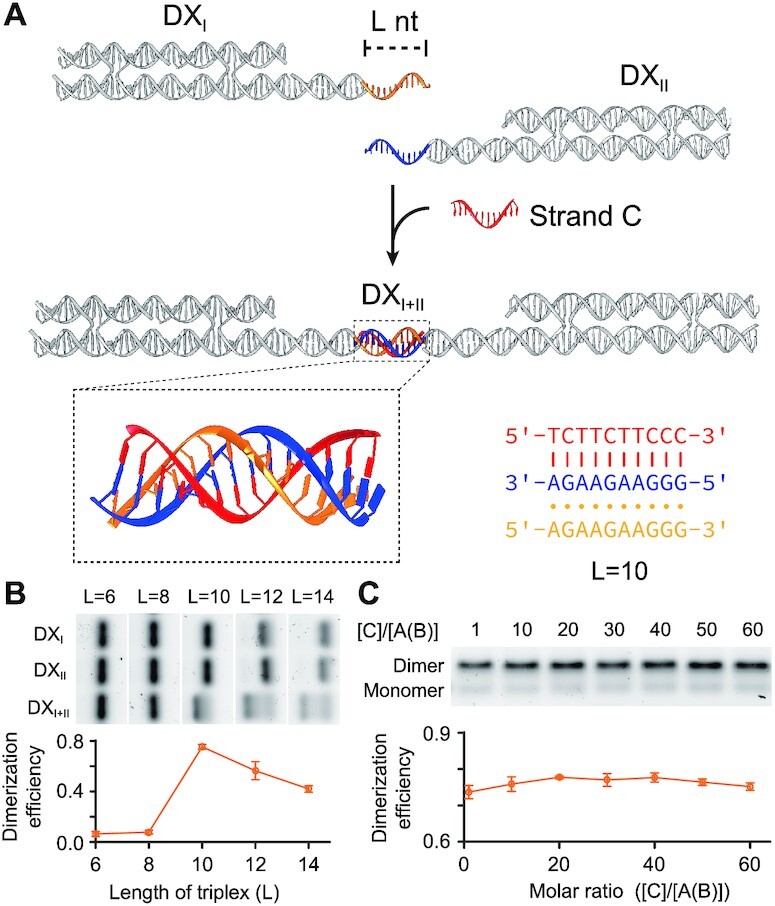
Ternary interaction on DX motifs. (**A**) Schematics of the DX structures appended with DNA triplex as ternary interaction. L: length (number of nucleobases) of each strand of the triplex structure. (**B**) Native AGE results (top panel) and statistical analysis (bottom panel) of the DX dimerization with different triplex length (L = 6, 8, 10, 12 or 14). (**C**) Native AGE results (top panel) and statistical analysis (bottom panel) of the DX dimerization (10-bt triplex) with excessive third strand C (molar ratio of [C] : [A(B)] = 1:1, 10:1, 20:1, 30:1, 40:1, 50:1 or 60:1).

To further examine the feasibility of triplex ternary interaction, we then investigated the formation of DNA triplex based on DX motifs. By applying two polypurine strands of the DNA triplex (A and B) to two different DX motifs (DX_I_ and DX_II_, Figure 2A), the third polypyrimidine strand (C) served as the modulator to initiate the interaction among these three components and resulted in dimeric DX structure (DX_I+II_). With DX motifs, we again found the 10-bt triplex segment showing an optimal binding performance (dimerization efficiency at ∼75% as shown in Figure 2B). Interestingly, with 12-bt or 14-bt triplex, the dimerization efficiency dropped substantially (∼56% for 12 bt and ∼42% for 14 bt, in Figure 2B). Our results are consistent with the findings in an earlier study (33) on the stability of antiparallel triplexes of different lengths. Presumably, the strong stacking interactions between adjacent nucleobases in polypurine strands might result in a conformation that is not conducive to the formation of reverse Hoogsteen base pairing. Besides, undesired structures such as GA duplexes and/or quadruplexes of particular sequences might also form, thus affect the binding performance of the triplex structure. Although triplex sequences used in this study were optimized by decreasing the guanine content (≤50%) and limiting the number of consecutive guanines (≤3), it was still challenging to completely rule out the undesired structures. From a practical point of view, we used this 10-bt triplex design for following DNA nanostructure systems.

In our design of ternary interaction, the polypyrimidine strand (strand C) served as the modulator which directly bound to only one polypurine strand through Watson-Crick hydrogen bond and successively triggered the reverse Hoogsteen hydrogen bond between the two polypurine strands. As a consequence, this type of ternary interaction did not suffer from binding performance drop in high modulator (strand C) concentration, with dimerization efficiency remained 70–80% at a 60-fold strand C to strand A(B) ratio (lane 7 in Figure 2C). On the contrary, a bridging modulator (strand C) binds to both strands A and B in the common sandwich-type ternary interaction, which was presented in recent work of ternary DNA interaction (34). A weakened binding performance (dimerization efficiency dropped to ∼27%, Supplementary Figure S3) in the presence of excessive amount of the modulator was shown in our results for the sandwich-type interaction, due to the blocking from two independent binary interactions where strand C hybridized to A and B (C–A and C–B) respectively (35). The insensitivity to stoichiometry of our ternary interaction strategy based on DNA triplex brings in extra robustness for applications in which optimal binding stoichiometry is difficult to be identified (e.g. regulating bacteria cell assembly in the later part of this study).

Encouraged by results of employing DNA triplex as ternary interaction in DX system, we next set to implement this ternary interaction on DNA origami structures. Presumably, it would be more difficult for the structural units with high charge-density (e.g. DNA origami units) to self-assemble due to the strong electrostatic repulsion, and therefore adjustment of the valency number (V) of ternary interaction to tune the binding affinity would play an important role to tackle the assembly challenge. Taking that into consideration, we investigated in a series of designs with gradient valency numbers for the optimized assembly.

**Figure 3.**
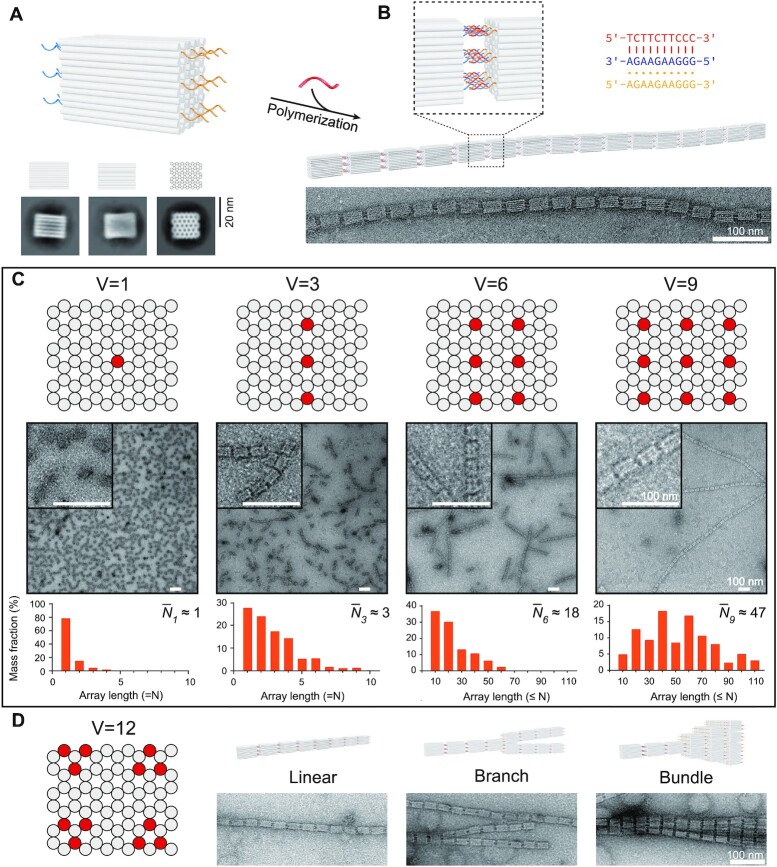
Ternary interaction on Janus DNA origami cuboids. Individual origami cuboids (**A**) assembled into nanofibrils (**B**) upon addition of modulator strand. Top panels: schematics; bottom panels: TEM 2D class average (A) and representative TEM image (B). (**C**) Schematics of cuboids with different valency numbers (V = 1, 3, 6 or 9) and corresponding results. Top panel: schematics of binding interface with handle locations highlighted; middle panel: TEM results and zoom-in views; bottom panel: histograms of mass fraction of resulted nanofibril lengths (component unit number) with the corresponding valency number. (**D**) Ternary interaction on cuboid origami with valency number of 12. Left panel: schematics of binding interface; right panel: schematics and TEM results of the representative polymerization configurations, such as linear, branched, and bundled polymerization. Scale bars: 100 nm.

Our Janus origami unit was designed as a cuboid of ∼32 × 20 × 16 nm with well-defined structural rigidity and Janus characters (20). The two opposite side faces of helical ends of the 72-helix honeycomb lattice served as binding interfaces, on which we placed the handles to dock the polypurine strands A and B of the triplex. By placing one to nine handles on each side face of the cuboid, customized valency number was presented (A_V_-Cuboid-B_V_, V = 1, 3, 6 or 9). As shown in native AGE analysis for the dimerization of origami cuboids with gradient valency (with A, B and C in equal molar ratio), the dimerization efficiency was in a positive correlation (Supplementary Figure S4). TEM results further confirmed the trend of dimer and trimer formation (Supplementary Figures S5–S7). Native AGE and TEM results also showed specific length distribution of polymerized origami nanofibrils among different valency numbers. Consistent with dimerization results, cuboid unit with valency number of 9 exhibited the best polymerization performance (evaluated by the average fibril length ($\overline {{N_9}}$) of ∼47 units, as shown in Figure 3C) and reached as long as ∼110 units, while the cuboids with lower valency number underperformed substantially ($\overline {{N_1}}$∼1, $\overline {{N_3}}$∼3 and $\overline {{N_6}}$∼18 for designs with respective valency number of 1, 3 and 6, as shown in Figure 3C and Supplementary Figures S8–S12).

Intriguingly, the cuboid design with valency number of 12 demonstrated different polymerization configurations such as linear, branched, and bundled polymerization (Figures 3D and S13). Presumably, an offset binding interface would be stable enough under this binding condition, thus branched and bundled polymerization configurations would be encouraged accordingly. Cuboid design with even higher valency number (i.e. V = 30) led to similar results with a variety of polymerization configurations (Supplementary Figures S14 and S15).

Apart from the aforementioned homopolymerization scheme [(A_V_-Cuboid-B_V_)]_n_, we also investigated in alternating copolymerization scheme [(A_V_-Cuboid-A_V_)-(B_V_-Cuboid-B_V_)]_n_ to construct origami nanofibrils, with TEM results showing the successful polymerization. The resulted nanofibrils were shorter ($\overline {N}$∼12, Supplementary Figure S16) than those from the homopolymerization scheme ($\overline {N}$∼47, Figure 3C), presumably due to the higher level of assembly complexity.

**Figure 4.**
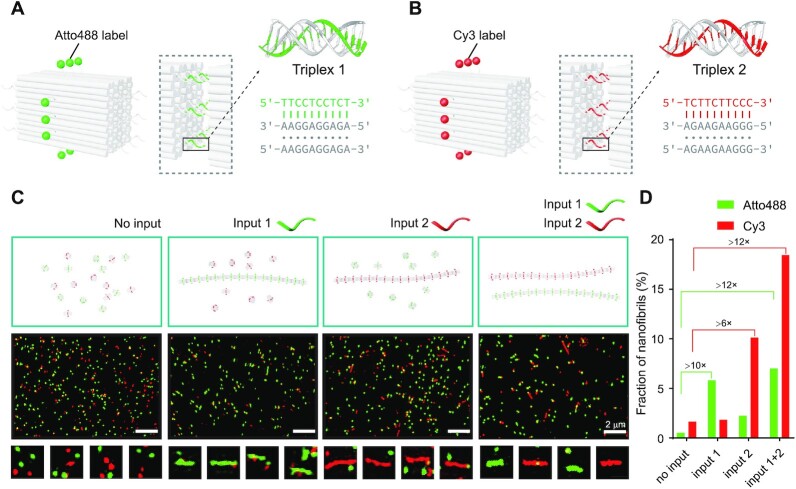
Selective signaling system based on the triplex-induced assembly of origami cuboids. (A, B) Schematics of specific origami cuboid assembly responsive to orthogonal signals. The production of nanofibrils decorated with Atto488 (**A**) and Cy3 (**B**) responsive to inputs 1 (green strand) and 2 (red strand) respectively. (**C**) Schematics of selection process (top panel) and the corresponding fluorescence microscopy images (middle panel, representative zoom-in views displayed at the bottom panel). Specific outputs responsive to no input, input 1, input 2 and both are shown from the leftmost to the rightmost. Scale bars: 2 μm. Zoom-in views scale: 1 μm^2^. (**D**) Histogram of the observable nanofibril fractions from all identifiable entities in confocal images. From left to right: no input, input 1, input 2 and both inputs.

Having demonstrated the feasibility of ternary interaction in controllable assembly of origami cuboids, we next sought to utilize this strategy in a selective signaling system to fish out orthogonal signals with the specific assembly as the readout. In such a system, two species of origami cuboids bearing two sets of triplex sequences can display exclusive assembly products according to respective signals (inputs 1 and 2 as shown in Figure 4A and B). Under atomic force microscopy imaging, the selective modulation of input 1 or input 2 was clearly illustrated, showing input-dependent formation of nanofibrils (Supplementary Figures S17-S20). To make the outputs distinguishable, we labeled these two species of origami cuboids with different fluorophores, one with Cy3 and the other with Atto488. In the absence of either input, the two species of cuboid units remained as discrete monomers with red or green dots presented under fluorescence microscopy. Red (with input 1) or green nanofibrils (with input 2) was presented with either input, while both red and green nanofibrils were presented at the same time with dual inputs (inputs 1 and 2, Figure 4C and S21–S24). To provide quantitative analysis of the assembled nanofibrils, we set a fluorescent intensity threshold to distinguish well-formed nanofibrils from individual units (details in Materials and Methods). The fraction of well-formed nanofibrils (above threshold) with positive inputs was at least 6-fold to that of the negative control as shown in Figure 4D, a clear-cut result of selective signaling.

**Figure 5.**
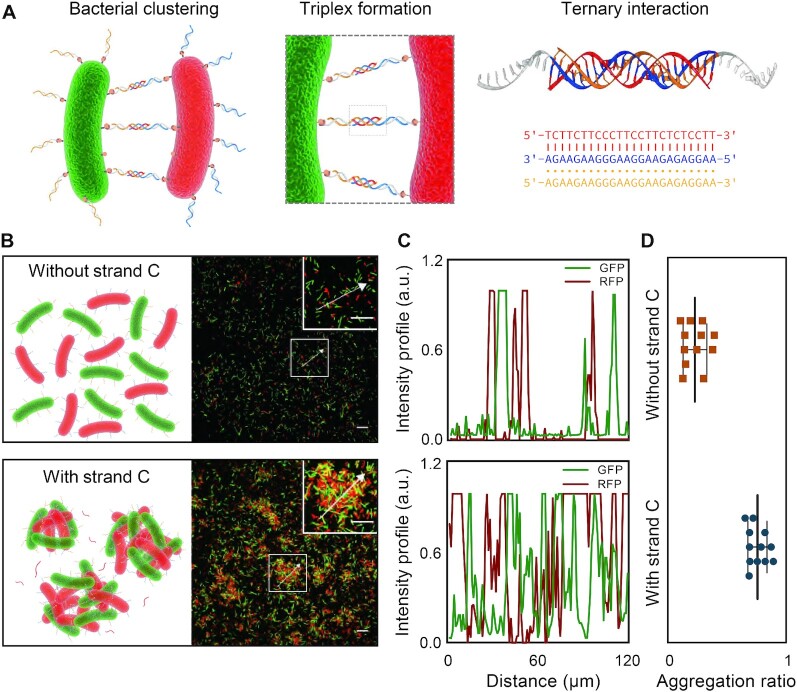
DNA ternary interaction mediated bacterial cell assembly. (**A**) Schematics of bacterial clustering via surface DNA modification and triplex-based ternary interaction. (**B**, **C**) Confocal microscopy images of DNA engineered GFP-*E. coli* and RFP-*E. coli* incubated without or with strand C and their co-responding intensity profile distribution. Scale bars: 20 μm. (**D**) Statistical analysis of aggregation ratio of bacteria assemblies without or with 24-bt DNA triplex formation. The error bars are the standard error from 12 images. *P* < 0.0001, Student's *t*-test.

In the last part of this study, we applied DNA triplex-based ternary interaction to regulate the organization of live bacterial cells into micrometer-scale aggregations. DNA strands were immobilized onto the surface of bacterial cells via reported metabolic labeling approach and sequential ring-strain promoted click chemistry (36,37) (Supplementary Figure S25). Two types of fluorescent bacteria cells, the RFP-*E. coli* and GFP-*E. coli*, were surface-engineered with corresponding polypurine strands (i.e. strands A and B) of DNA triplex respectively to regulate the clustering of bacteria cells (Figure 5A). Without modulator strand C, the mixed RFP-*E. coli* and GFP-*E. coli* (with strands A and B modification respectively) evenly dispersed, and no cell clustering was observed. However, after co-incubating these two types of bacteria with modulator strand C for 5 h, prominent bacterial cell clustering was observed under confocal microscope, indicating the formation of DNA triplex (Figure 5B).

To quantitatively evaluate the performance of triplex-based ternary interaction in organizing bacterial clusters, we introduced ‘aggregation ratio’, which was the area occupied by bacterial clusters (clusters were defined by those whose area > 15 μm^2^) divided by the total area occupied by all bacteria (38), to illustrate the degree of bacterial assembly. By optimizing the length of DNA triplex from 10 bt, 12 bt, 16 bt, 20 bt, to 24 bt, the 24-bt DNA triplex was found to induce the most prominent bacterial assembly, resulting in an average cluster size of ∼89 μm^2^ (Supplementary Figure S26). Presumably, unlike DNA origami surface of well-defined structure, the complex surface morphologies of bacteria cells might result in steric hindrance and non-specific interactions which in turn compromised the triplex binding. The lengthened triplexes of 24-bt might serve as a compensation to increase the efficiency of bacterial assembly. In the presence of the 24-nt modulator strand C, 76% of the bacteria were integrated into clusters and GFP/RFP intensity profile presented distinctly compact and interlaced peaks, which indicated the formation of high-density bacterial assembly. While only 21% of bacteria were found clustered in the absence of the modulator strand, most bacteria cells were dispersed and showed sporadic fluorescence intensity peaks (Figure 5C and D). The assembly of micrometer-scale bacterial clusters induced by DNA triplex further demonstrated the generality and robustness of our ternary interaction approach, indicating its potential to regulate other complex biological systems (39).

## DISCUSSION

Taking advantage of the sequence dependent and programmable DNA triplex, we have implemented a triplex-based ternary interaction for hierarchical assembly in this work. The robust assembly strategy has enabled the controllable construction of a variety of DNA assemblies from preformed Janus DNA origami units, based on which we also established a signaling system with assembly behaviors responsive to specific signals. The triplex strategy has been further demonstrated in organizing individual microbial cell into live clusters beyond micrometer scale, not only indicating the potential to apply this strategy to maneuver complex biological processes to cellular level and beyond, but also providing a practical route toward dynamic regulation of the formation of artificial cell community that may be useful for potential antimicrobial therapies.

Despite the promises, the precision level of controllability for DNA ternary interaction is still limited. For example, the length distribution of resulted nanofibrils from origami cuboids is wide and the polymerization configurations are mixed instead of distinct for origami cuboid designs of high interaction valency numbers. It is also challenging to achieve directional and anisotropic control of the triplex-induced assembly process of bacterial cells because of the homogeneous surface modification and the moving nature. However, we believe lessons could be learned from Nature to improve the controllability of the self-assembly processes. Efficient and precise ternary interactions are commonly presented among native biomolecules to drive many sophisticated cellular activities (40–44). With the knowledge learned from natural systems involving multi-component interactions and modulations, we believe more research opportunities would emerge to enable better controllability in spatiotemporal regulation of chemical and biological activities for synthetic systems.

## DATA AVAILABILITY

Designs and DNA sequences of all constructs are available in Supplementary Figures S27–S29 and Supplementary Tables S1–S13 in the Design and Sequences section of Supplementary Information. All data needed to evaluate the conclusions in the paper are present in the paper and/or Supplementary Information.

## Supplementary Material

gkac478_Supplemental_File
