# Supplementary material for: Controllable assembly of synthetic constructs with programmable ternary DNA interaction
Source: Nucleic Acids Res. 2022 Jun 17;50(12):7188–96. doi: 10.1093/nar/gkac478 (PMC9262601; doi:10.1093/nar/gkac478)
Supplement: gkac478_Supplemental_File [file gkac478_supplemental_file.pdf]

Controllable assembly of synthetic constructs with programmable ternary DNA interaction

Huangchen Cui[1,†], Tianqing Zhang[1,†,*], Yuhan Kong[2,†], Hang Xing[2,*], Bryan Wei[1,*]

[1]School of Life Sciences, Tsinghua University-Peking University Center for Life Sciences, Center for Synthetic and Systems Biology, Tsinghua University, Beijing 100084, China
[2]Institute of Chemical Biology and Nanomedicine; State Key Laboratory of Chemo/Biosensing and Chemometrics; Hunan Provincial Key Laboratory of Biomacromolecular Chemical Biology; College of Chemistry and Chemical Engineering; Hunan University, Changsha, 410082, China
[†]These authors contributed equally to this work.
[*]Corresponding Author. Email: bw@tsinghua.edu.cn (B.W.), hangxing@hnu.edu.cn (H.X.) and ztq17@mails.tsinghua.edu.cn (T.Z.).

This PDF file includes:

Supplementary Figures S1 to S26

Supplementary Notes 1 and 2

Design and Sequences

Supplementary Figures

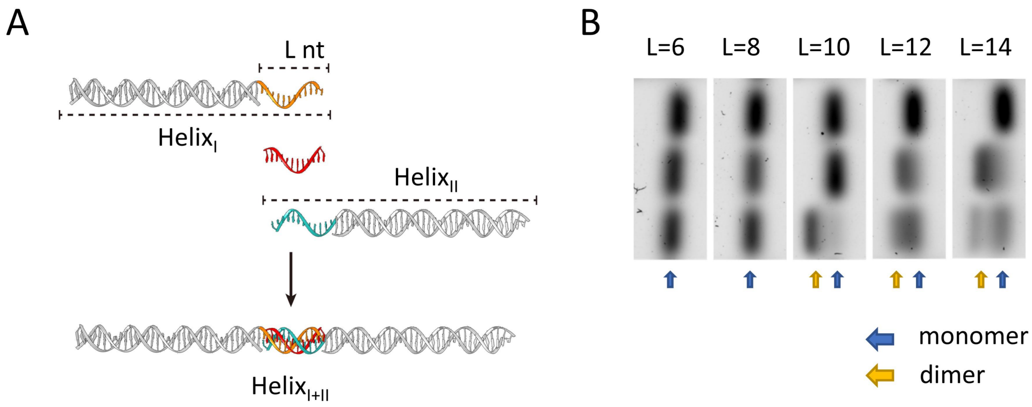

Figure S1. Ternary interaction on DNA duplex structure. A, Schematics of the duplex structures appended with DNA triplex as ternary interaction. **B**, Native AGE results of the duplex dimerization with different triplex length (L=6, 8, 10, 12, or 14). Monomers and dimers are marked with blue and yellow arrows, respectively.

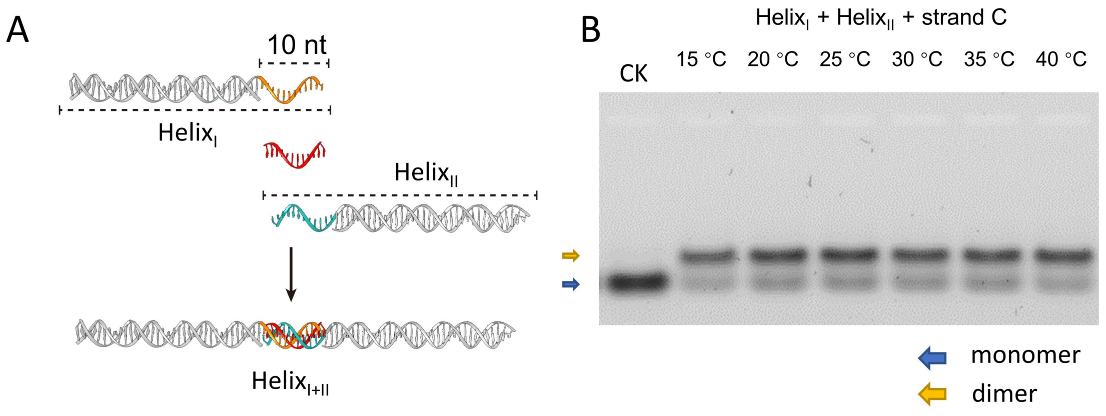

Figure S2. Stability test of the triplex based ternary interaction on DNA duplex structure. A, Schematics of the duplex structures appended with DNA triplex (10 bt) as ternary interaction. **B**, Native AGE results of the duplex dimerization in gradient treatment. Monomers and dimers are marked with blue arrows and yellow arrows, respectively.

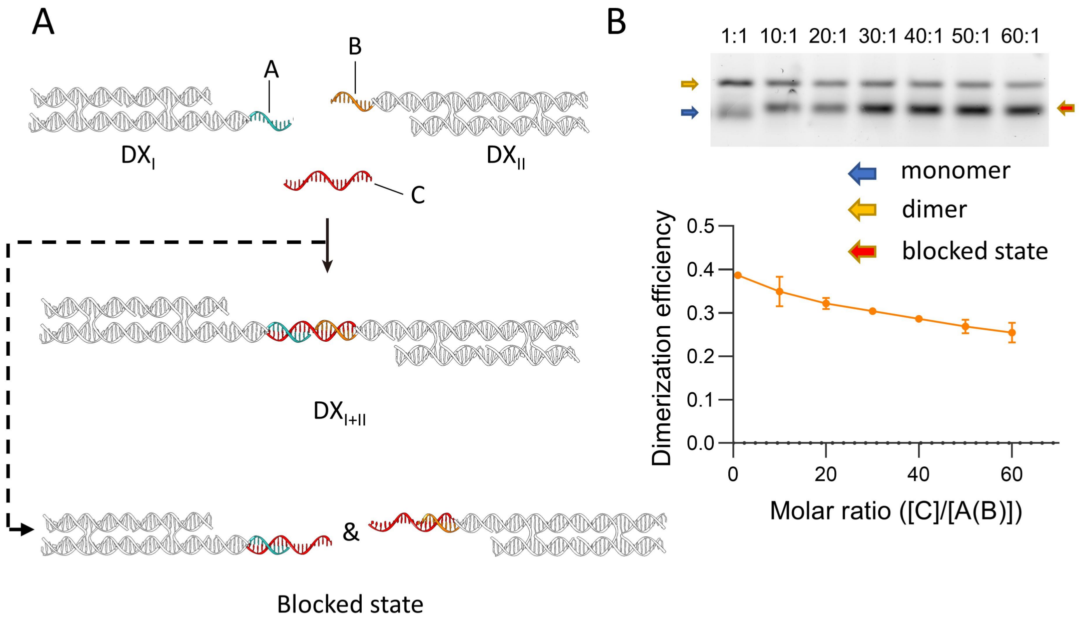

Figure S3. The common sandwich-like ternary interaction on DNA DX structure. A, Schematics of the DX structures appended with sandwich-like ternary interaction. As a comparison to triplex-based ternary interaction, we designed two 10-nt single stranded arms on the helical ends of two DX structures, respectively. A 20-nt single stranded modulator serving as the bridging component to link the two arms together, resulting in dimerized DX structure. **B**, Native AGE results (top panel) and statistical analysis (bottom panel) of the DX dimerization (with sandwich-like ternary interaction) with excessive third strands C (molar ratio of [C]/[A(B)] = 1:1, 10:1, 20:1, 30:1, 40:1, 50:1, or 60:1). Monomers and dimers are marked with blue and yellow arrows, respectively. For the common sandwich-type ternary interaction at a high strand C to strands A(B) ratio (e.g., 60-fold), blocked components (A-C and B-C) formed due to individual copies of C bound to A and B separately, which prohibited the formation of the dimeric DX structure.

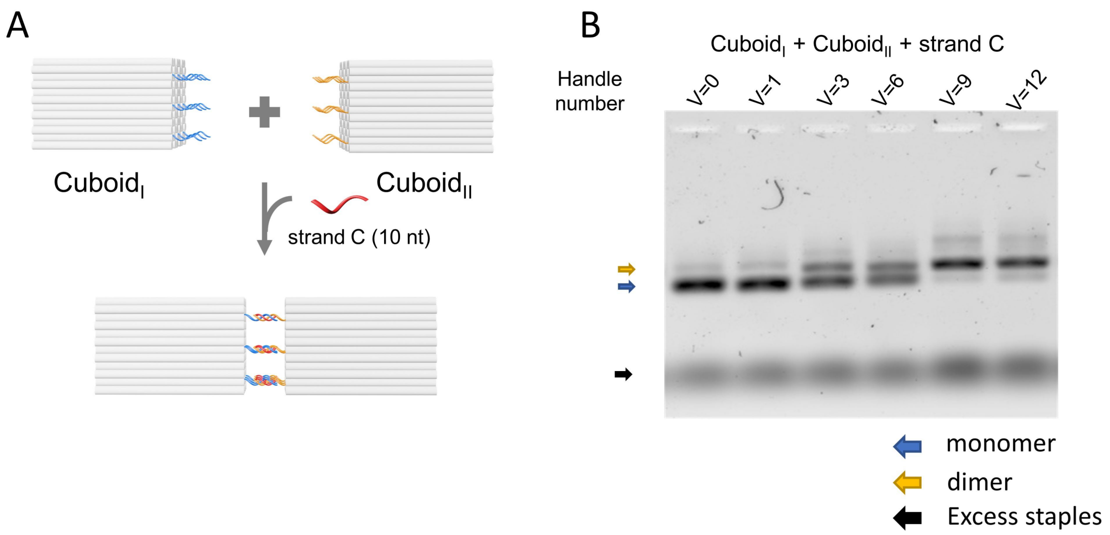

Figure S4. Ternary interaction on DNA origami cuboids. A, Schematics of the DNA origami cuboids appended with triplex-based ternary interaction. **B**, Native AGE results of the origami dimerization with different valency numbers. Monomers, dimers and excess staples are marked with blue, yellow and black arrows, respectively. We note the formation of nonspecific products of multimers (e.g., sample of V=0), and it is presumably due to the nonspecific base pairing or base stacking.

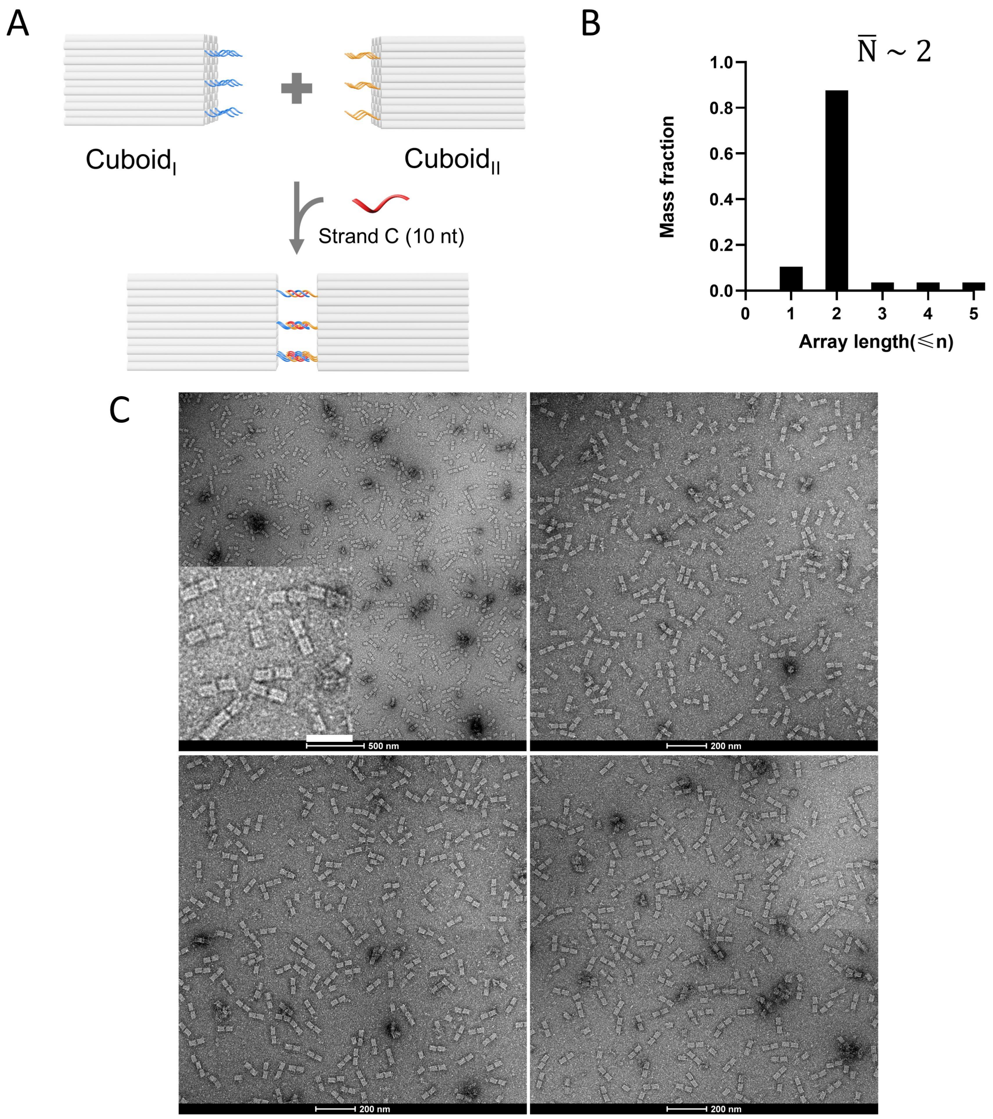

Figure S5. Dimerization of DNA origami cuboids via triplex-based ternary interaction. **A**, Schematics of the DNA origami cuboids appended with triplex-based ternary interaction (with length of 10 nt). **B**, Statistical analysis from TEM results (**C**) of the origami cuboid dimerization (valency number of 9).

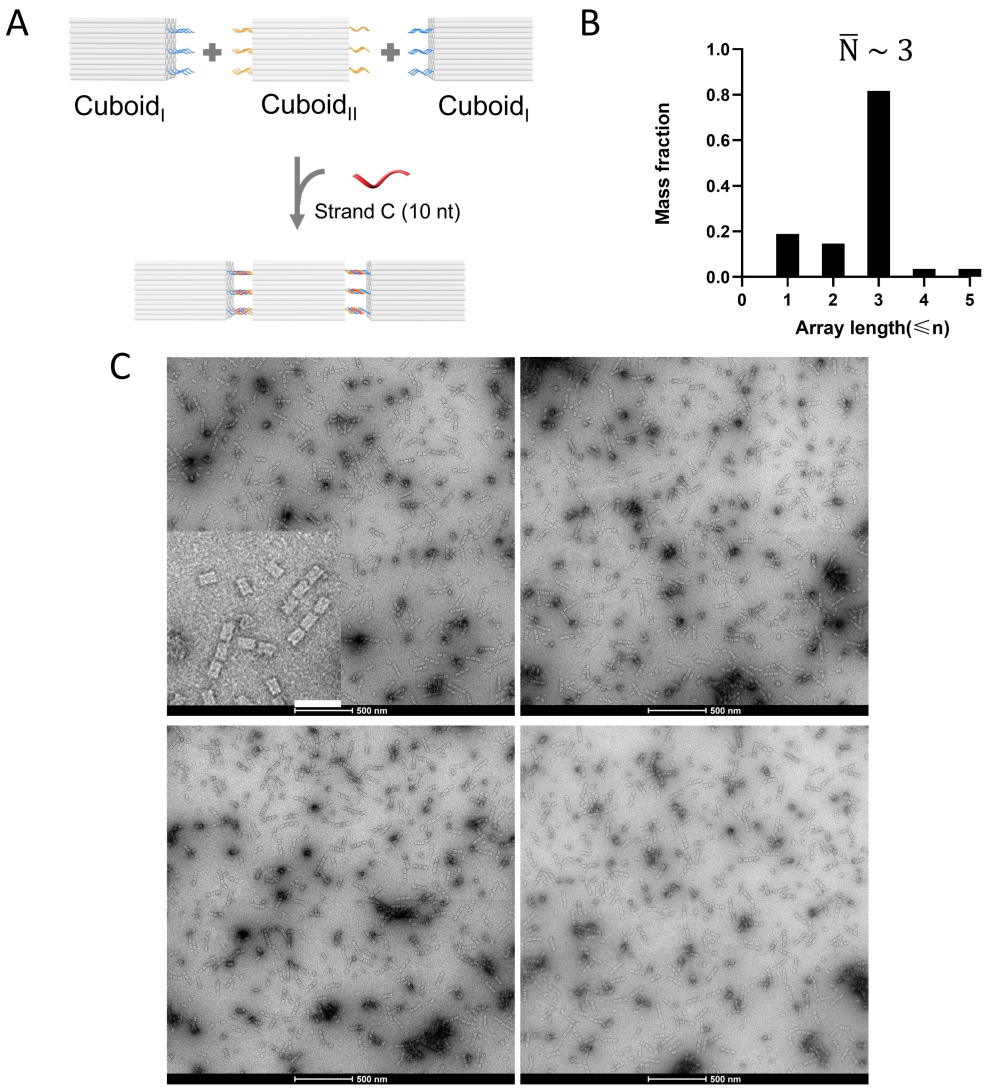

Figure S6. Trimerization (Cuboid$_I$-Cuboid$_{II}$-Cuboid$_I$) of DNA origami cuboids via triplex-based ternary interaction. **A**, Schematics of the DNA origami cuboids appended with triplex-based ternary interaction (with length of 10 nt). **B**, Statistical analysis from TEM results (**C**) of the origami cuboid trimerization (valency number of 9).

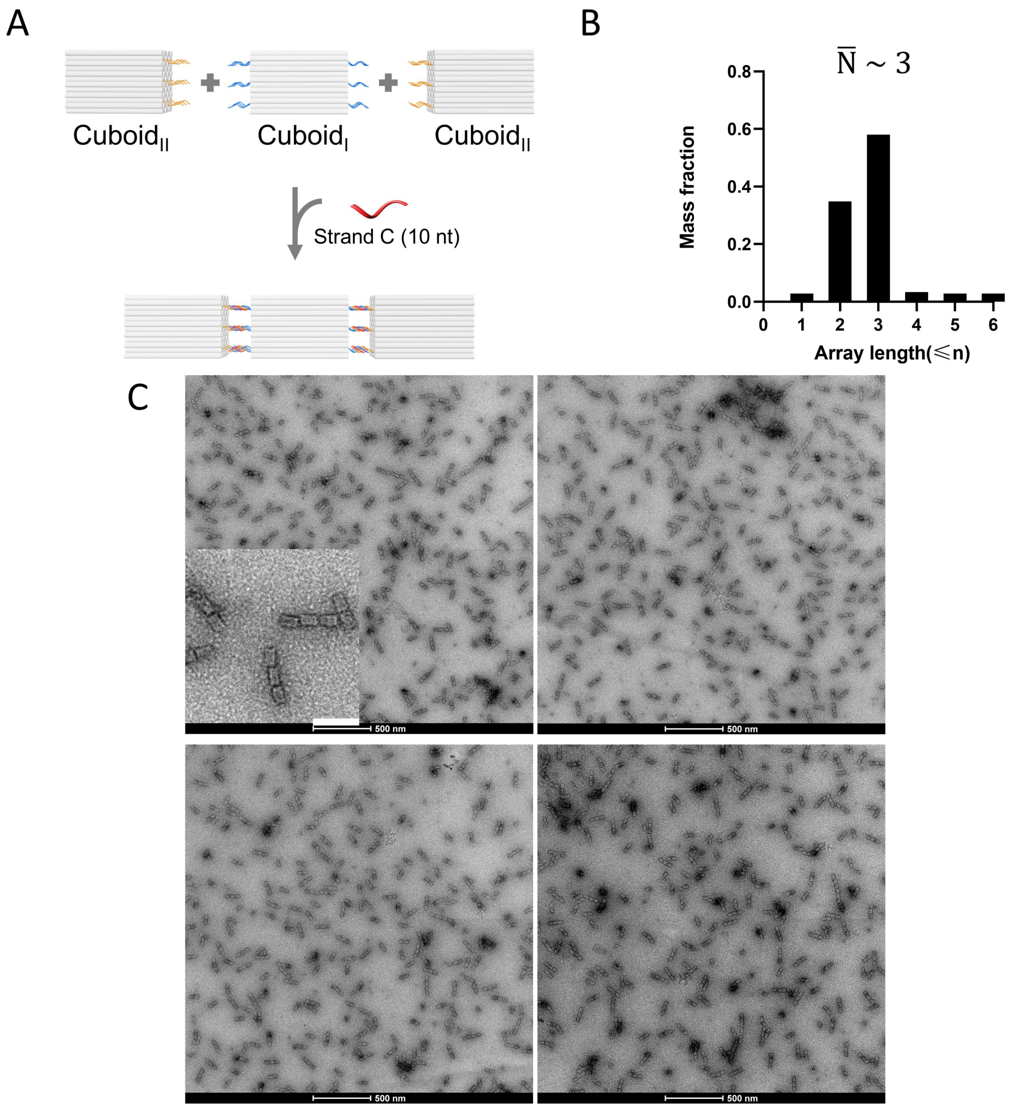

Figure S7. Trimerization (Cuboid$_{II}$-Cuboid$_{I}$-Cuboid$_{II}$) of DNA origami cuboids via triplex-based ternary interaction. **A**, Schematics of the DNA origami cuboids appended with triplex-based ternary interaction (with length of 10 nt). **B**, Statistical analysis from TEM results (**C**) of the origami cuboid trimerization (valency number of 9).

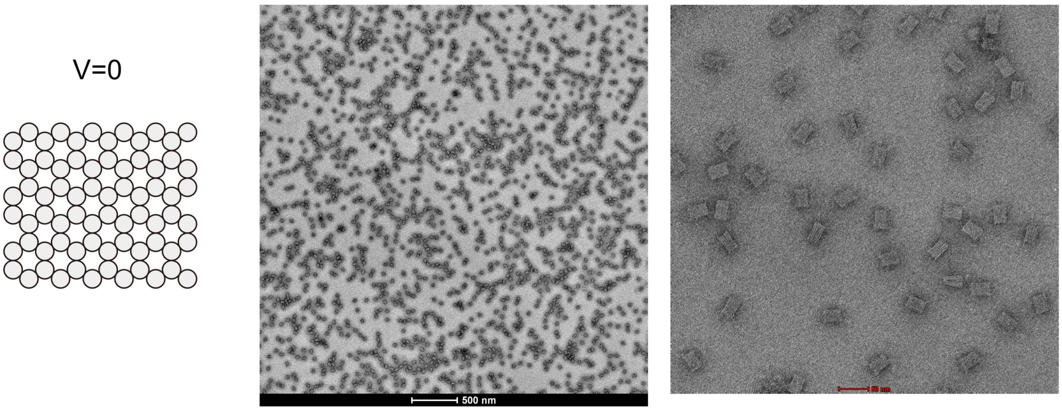

Figure S8. Schematics of cuboids (valency number of 0) and the representative TEM results.

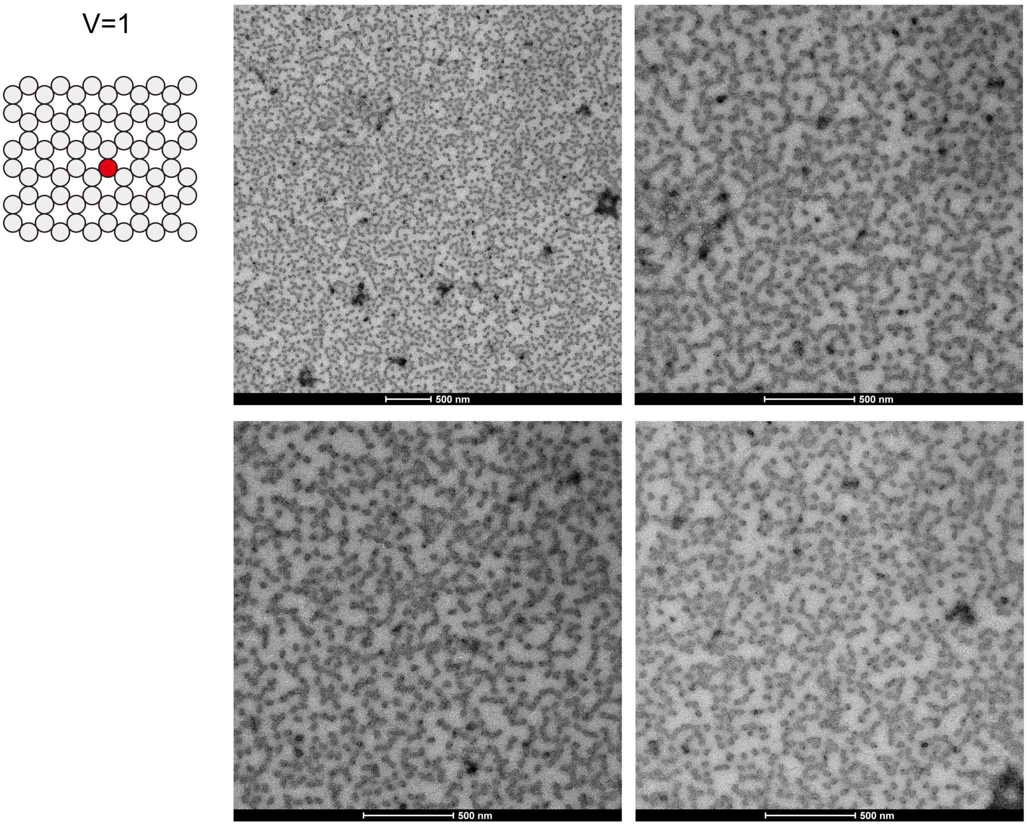

Figure S9. Schematics of cuboids (valency number of 1) and the representative TEM results.

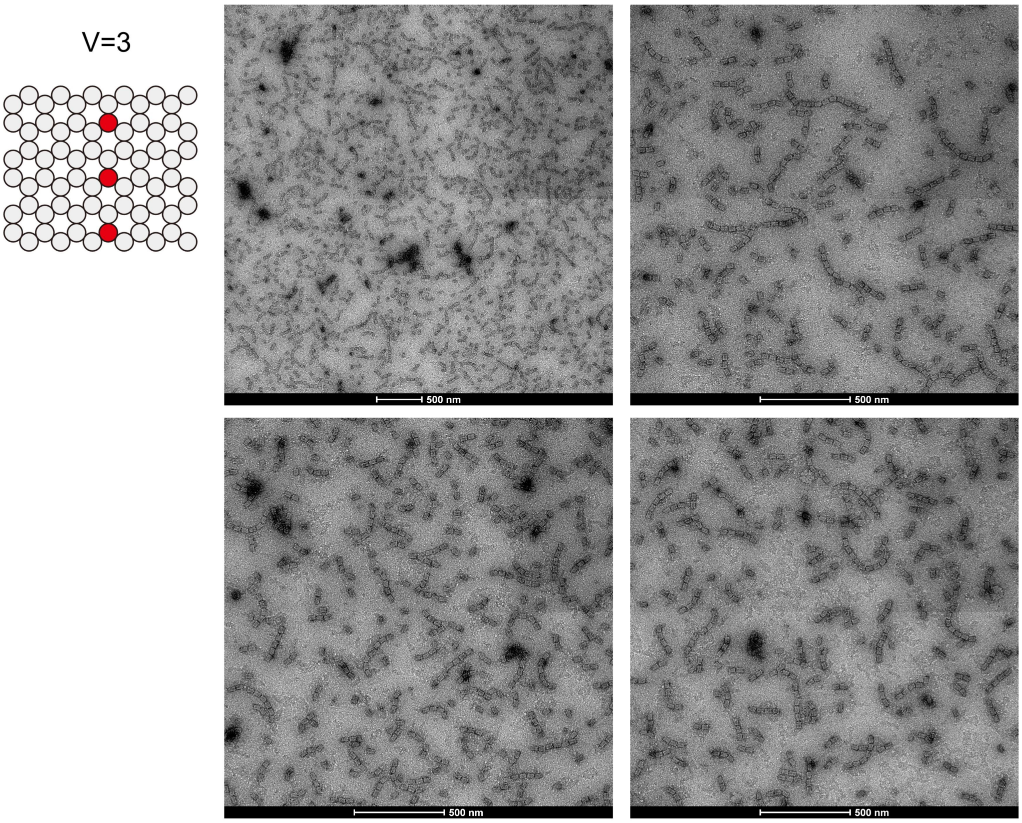

Figure S10. Schematics of cuboids (valency number of 3) and the representative TEM results.

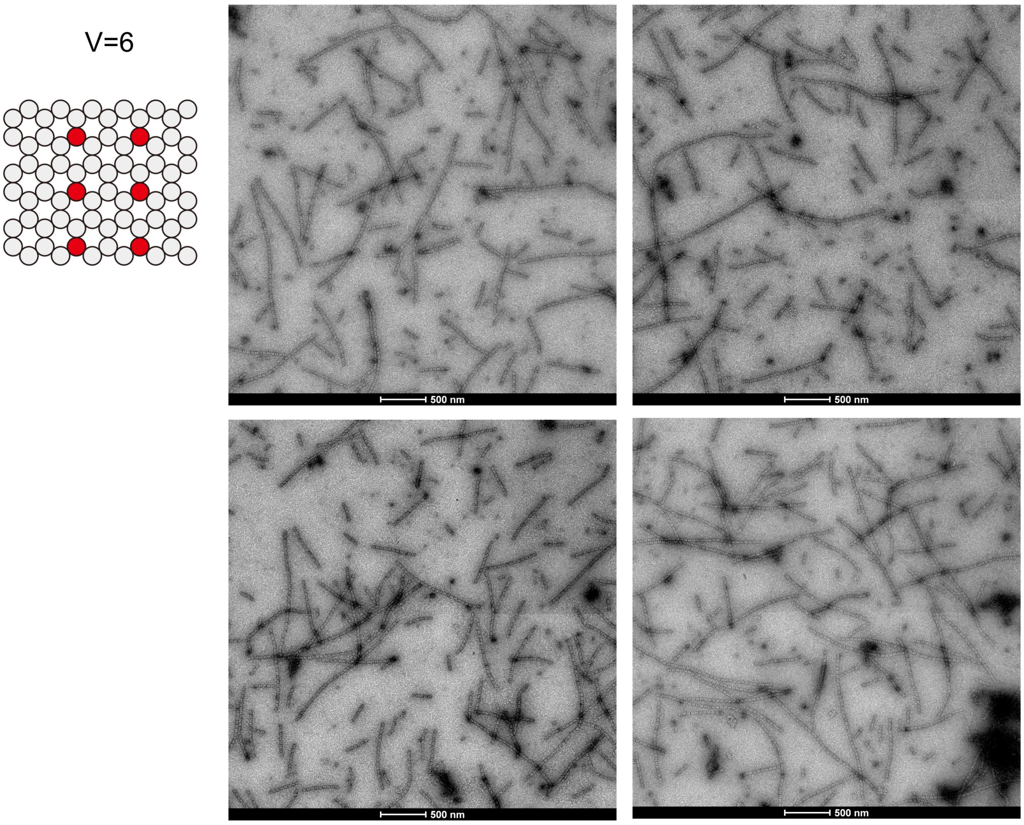

Figure S11. Schematics of cuboids (valency number of 6) and the representative TEM results.

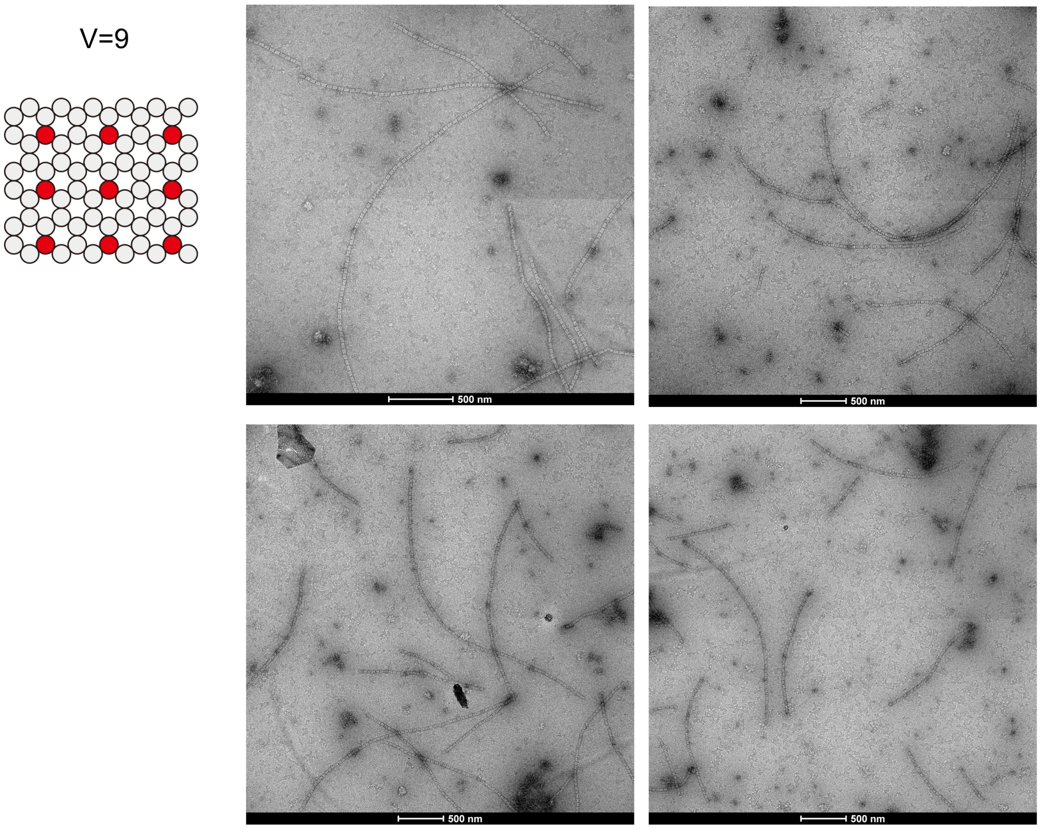

Figure S12. Schematics of cuboids (valency number of 9) and the representative TEM results.

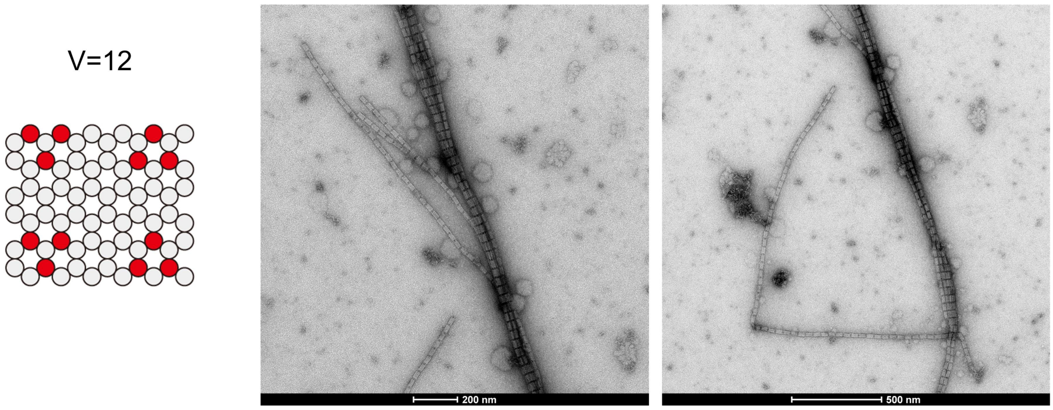

Figure S13. Schematics of cuboids (valency number of 12) and the representative TEM results.

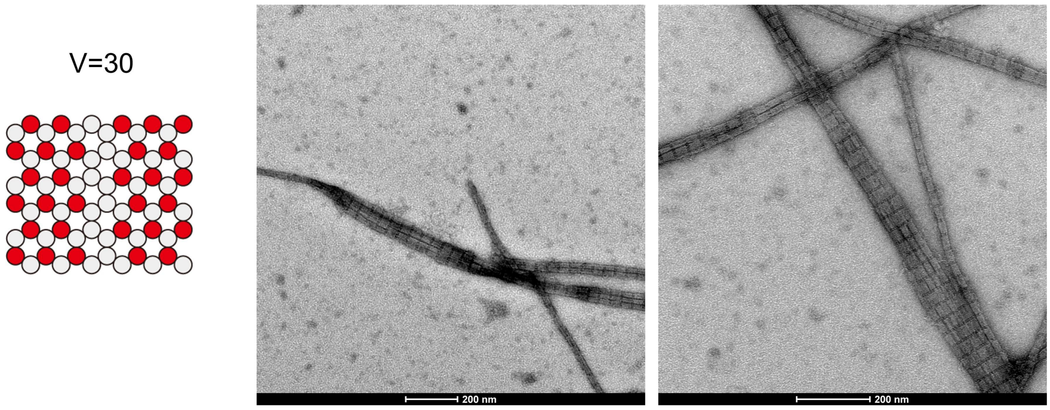

Figure S14. Schematics of cuboids (valency number of 30) and the representative TEM results.

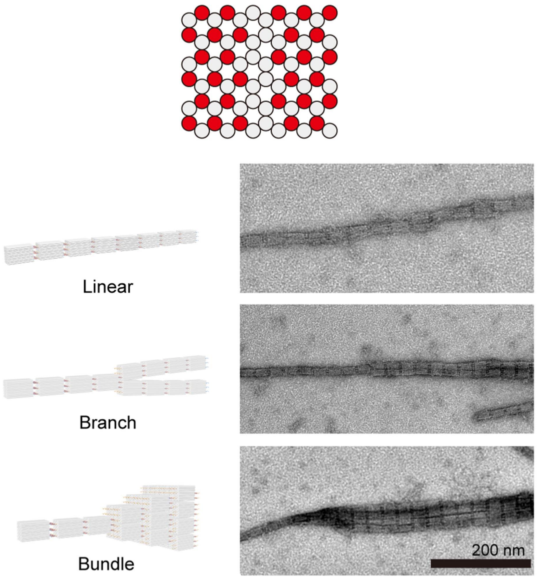

Figure S15. Ternary interaction on cuboid origami (valency number of 30). Top panel: schematics of binding interface; bottom panel: schematics and TEM results of the representative polymerization configurations, such as linear, branched, and bundled polymerization.

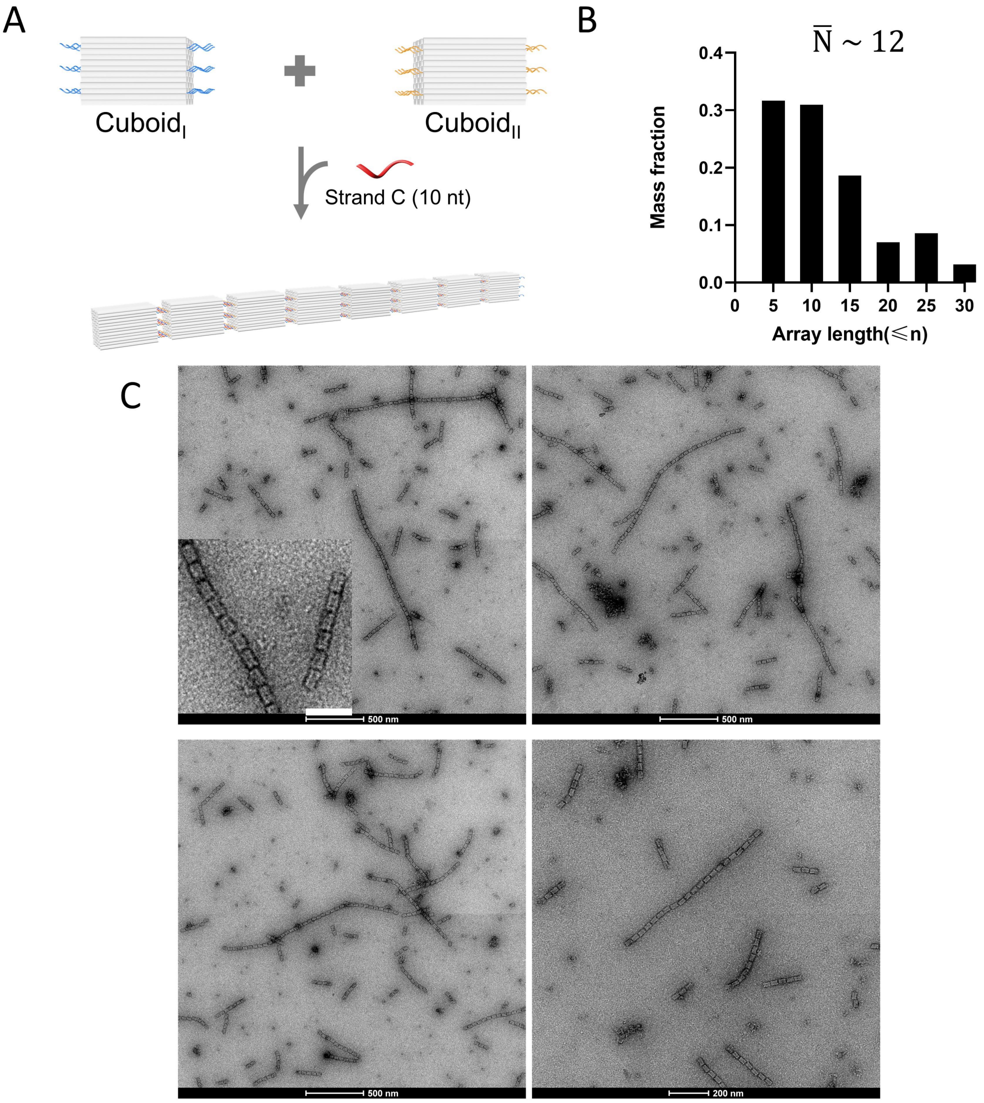

Figure S16. Alternating copolymerization ($[\text{Cuboid}_{I}\text{-Cuboid}_{II}]_n$) of DNA origami cuboids via triplex-based ternary interaction. A, Schematics of the DNA origami cuboids appended with triplex-based ternary interaction (with length of 10 nt). **B**, Statistical analysis from TEM results (**C**) of the origami cuboid alternating copolymerization (valency number of 9).

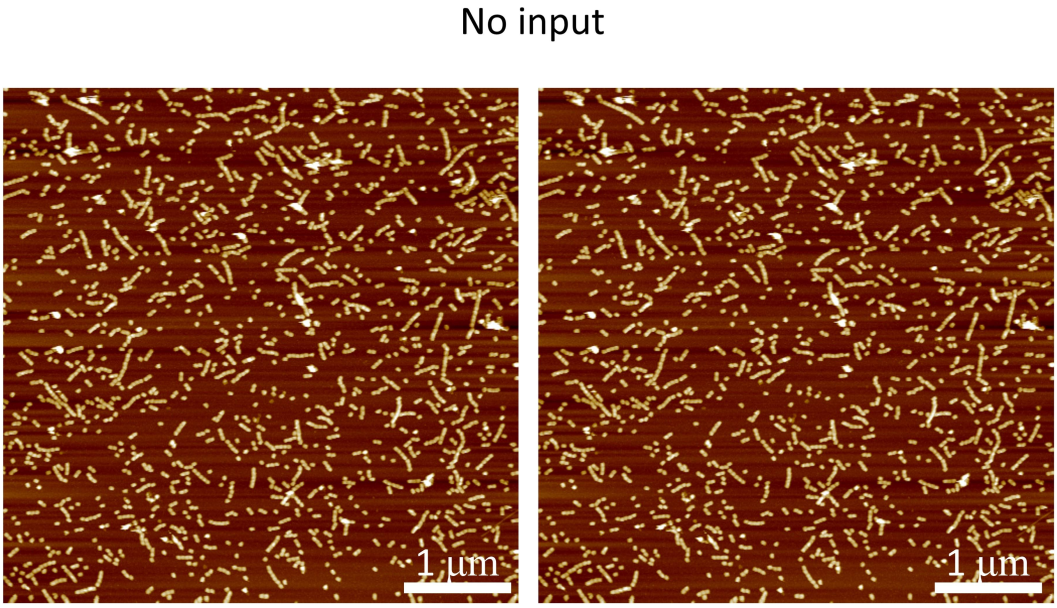

Figure S17. Representative atomic force microscopy (AFM) images of the selective signaling system without input. In our selective signaling system, two types of origami cuboids were designed with the same valency number (V=9). When they were mixed at the stoichiometric equivalent amount of 1:1 without any inputs, there were no nanofibrils in AFM images.

Input 1

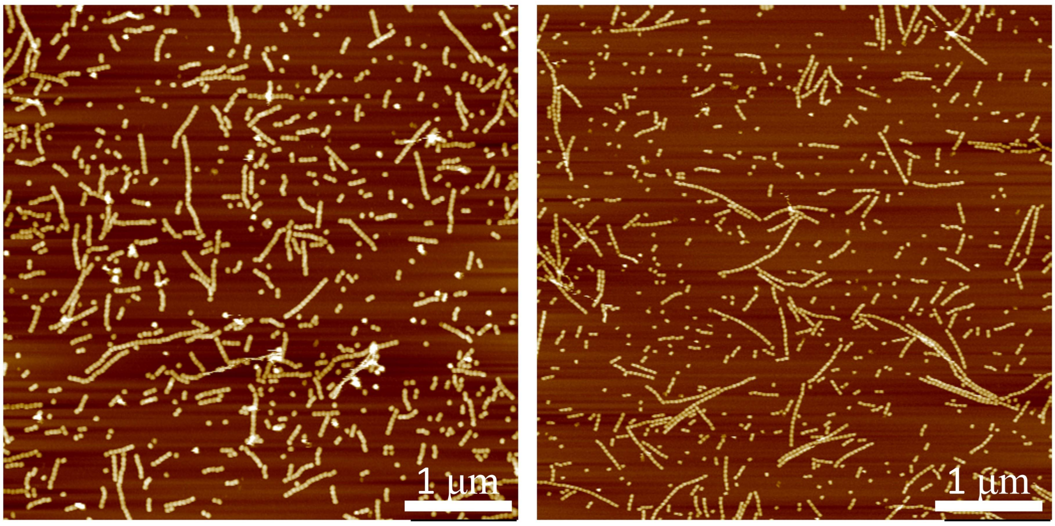

Figure S18. Representative AFM images of the selective signaling system with input 1. With input 1, individual units and assembled nanofibrils co-exist in AFM images, demonstrating the modulatory role of input 1 for triplex 1.

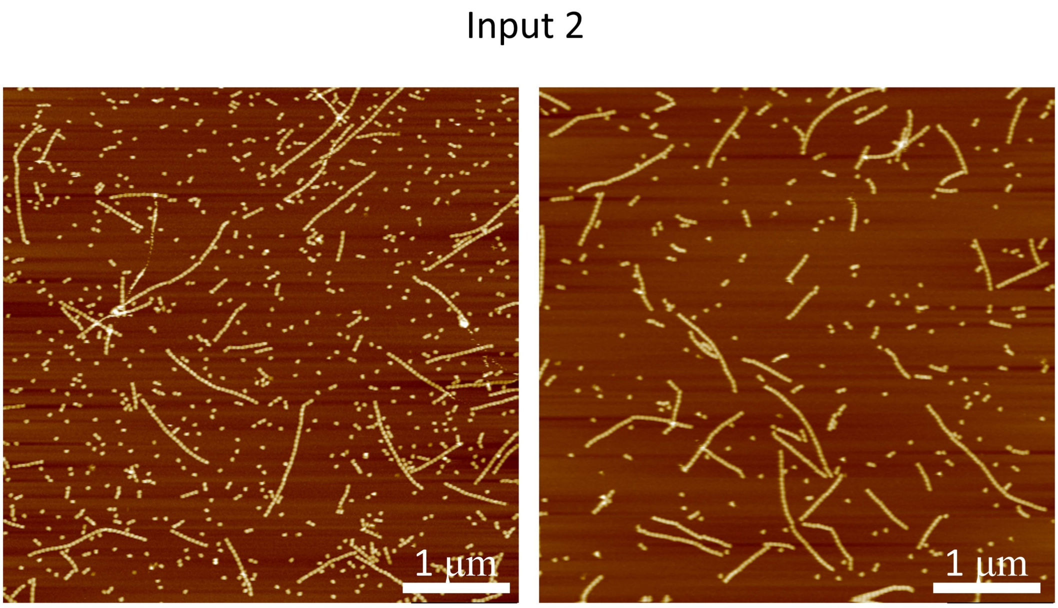

Figure S19. Representative AFM images of the selective signaling system with input 2. With input 2, individual units and assembled nanofibrils co-exist in AFM images, demonstrating the modulatory role of input 2 for triplex 2.

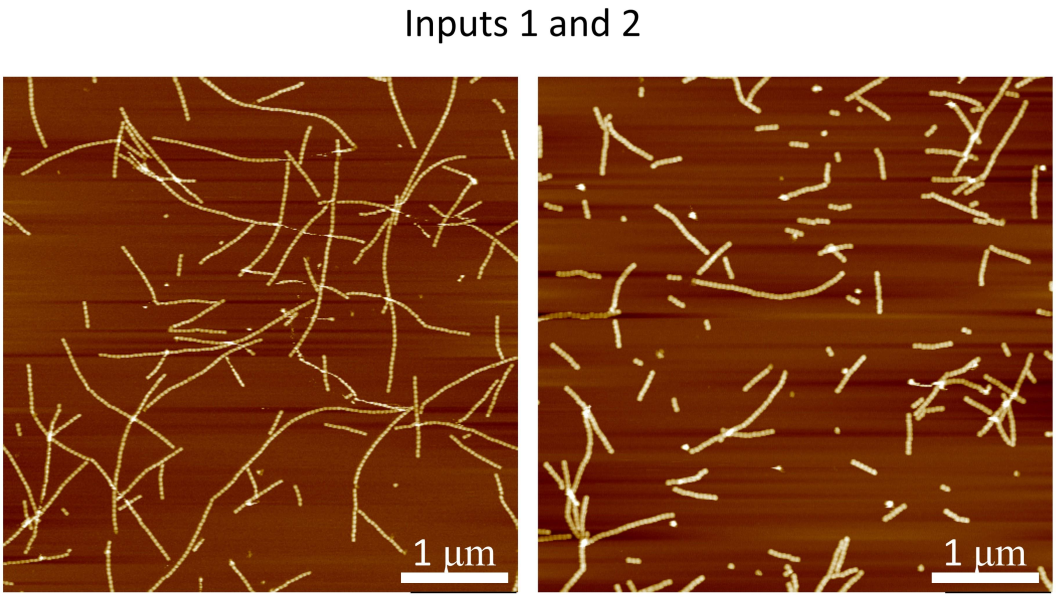

Figure S20. Representative AFM images of the selective signaling system with input 1 and 2. With input 1 and 2, most of the units assembled into nanofibrils in AFM images, demonstrating the modulatory role of input 1 for triplex 1 and input 2 for triplex 2 simultaneously.

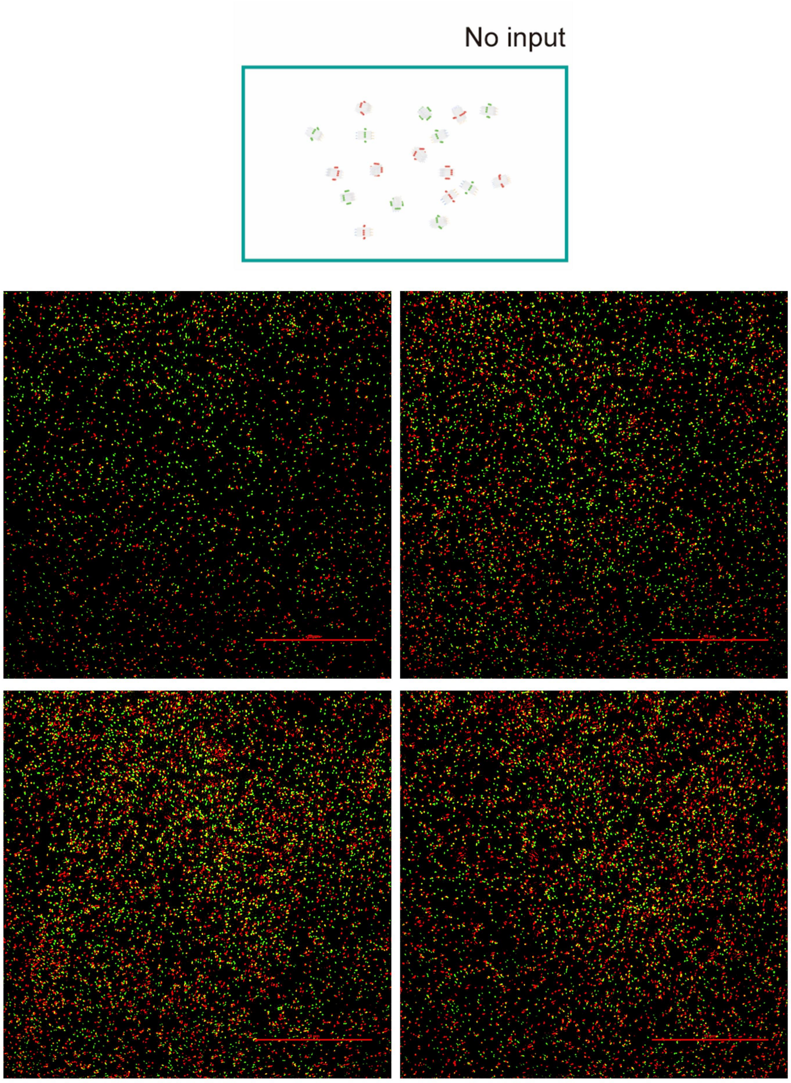

Figure S21. Schematics of the selective signaling system without input and representative fluorescence microscopy images. Scale bars: 10 μm.

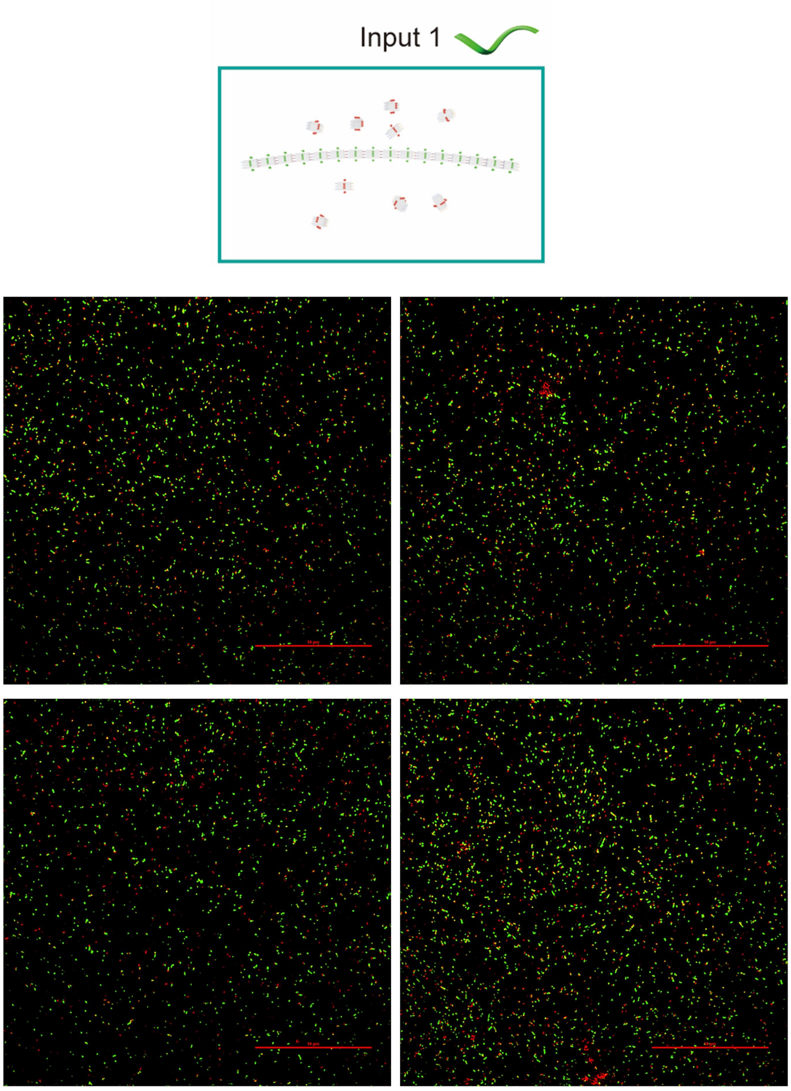

Figure S22. Schematics of the selective signaling system with input 1 and representative fluorescence microscopy images. Scale bars: 10 μm.

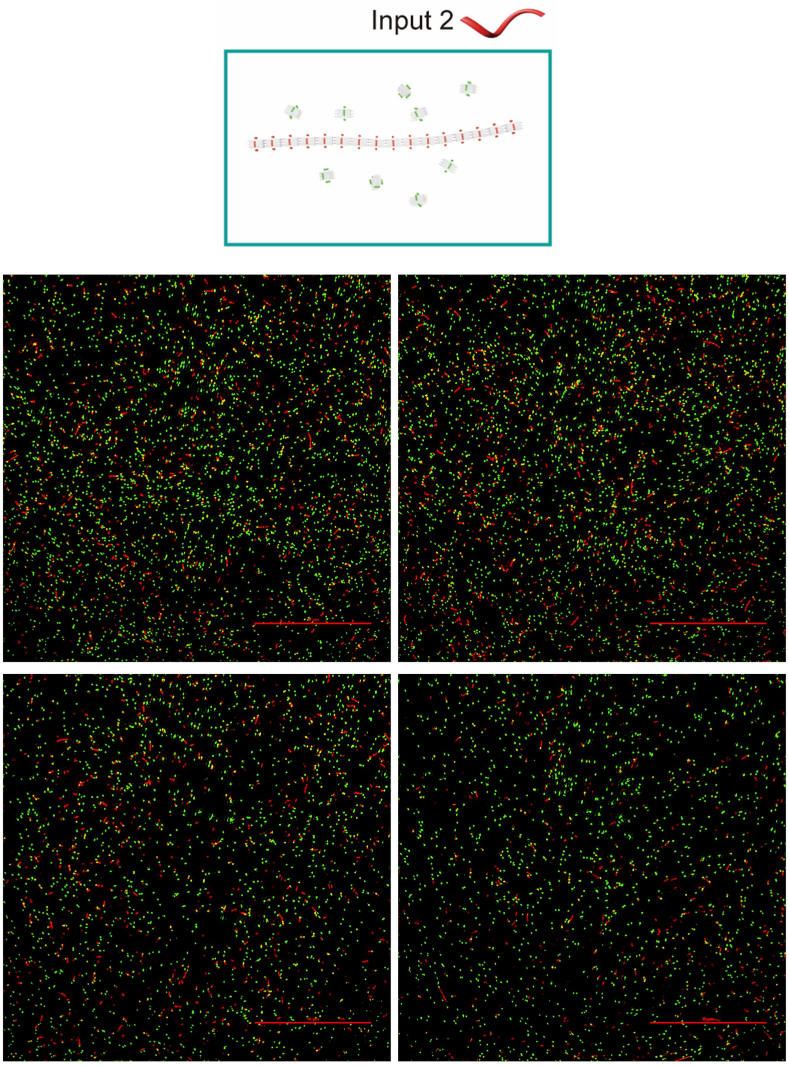

Figure S23. Schematics of the selective signaling system with input 2 and representative fluorescence microscopy images. Scale bars: 10 μm.

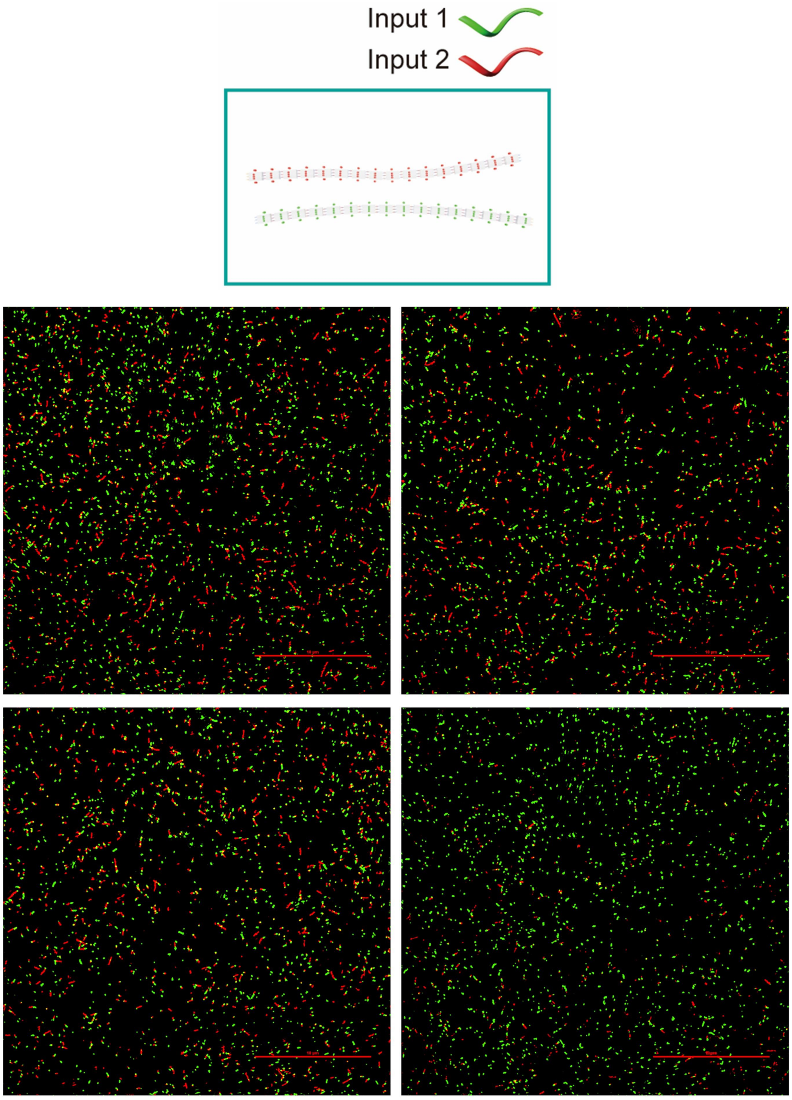

Figure S24. Schematics of the selective signaling system with input 1 as well as input 2 and representative fluorescence microscopy images. Scale bars: 10 μm.

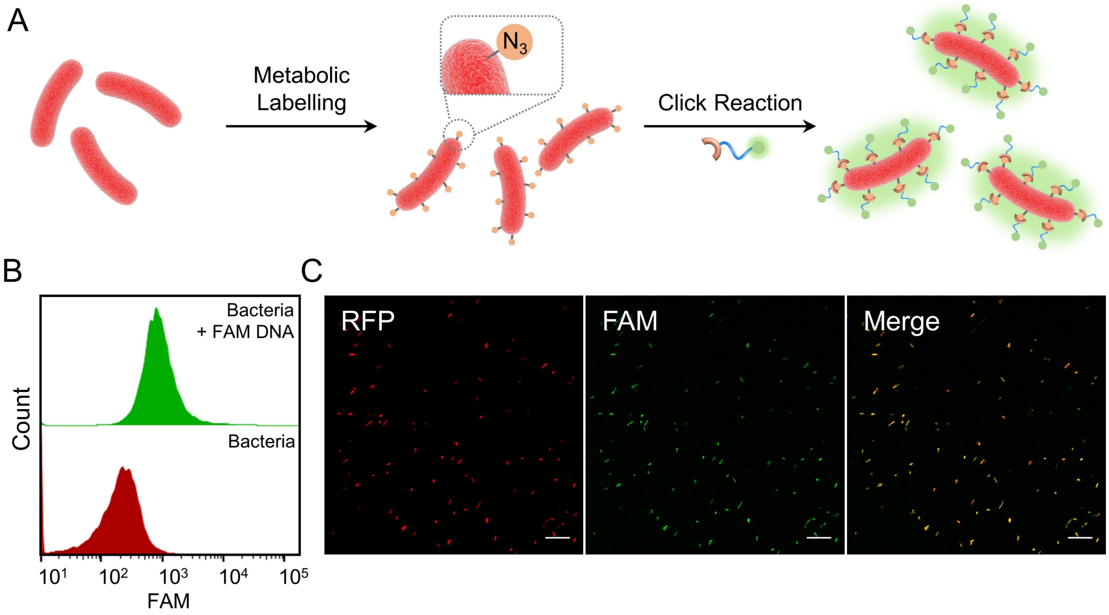

Figure S25. A. Schematics of DNA engineering on bacterial surface via metabolic labelling and click reaction. **B**. Flow cytometry showed that azide-*E. coli*-RFP surface was labelled by DBCO-DNA-FAM via click reaction. **C**. Confocal images indicated well colocalization of RFP signal from bacterial cell and FAM signal from DNA strands. Scale bars: 20 μm.

Supplementary Note 1

In order to identify the feasibility of DNA strands modified on bacterial surface, *E. coli*-RFP cells metabolically labelled with azide ligands[1] were co-incubated with DNA strands (5' and 3' were modified with DBCO and FAM, respectively). DBCO groups function as reactive handles for the covalent triazole linkage with azide-bacteria (Figure S25 A). The enhanced shift of FAM signal peaks of DBCO-DNA-FAM treated azide-*E. coli*-RFP indicated the successful DNA modification of bacterial surface (Figure S25 B). Consistent with flow cytometry results, confocal images of treated group exhibited well colocalization of RFP and FAM signal, which further indicated a high DNA ligation efficiency onto bacterial cell surface via metabolic engineering and click reaction. (Figure S25 C).

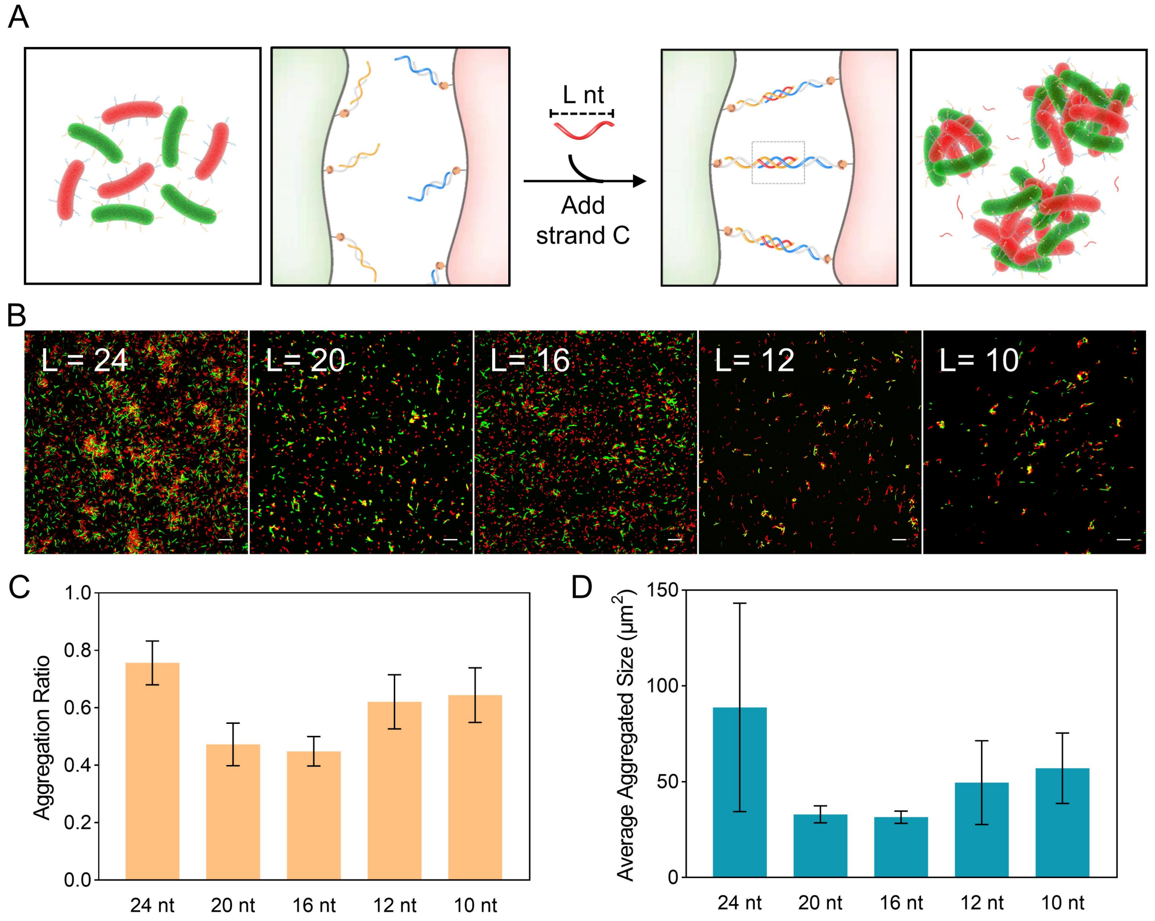

Figure S26. A. Schematics of ternary interaction mediating GFP and RFP bacterial cells assembly using different lengths of triple helix. **B**. Confocal images show different assembly behaviors of *E. coli*-GFP and *E. coli*-RFP induced by different triple helices (L=24, 20, 16, 12 or 10). DNA modified *E. coli*-GFP and *E. coli*-RFP were mixed in a 1:1 ratio (OD600=0.5) and incubated for 5 h. Scale bars: 20 μm. **C**. Statistical analysis of aggregation ratio of bacteria assemblies mediated by different lengths of triplex. The aggregation ratio indicates the area of bacterial assemblies with an area >15 μm^2 divided by the area occupied by all bacteria in one image. **D**. Statistical analysis of average aggregated size of each group. The error bars in C and D are the standard error from 10 images.

Supplementary Note 2

Long and rigid structure will allow polypurine ends stretching out from bacterial rough surface to enable accessible DNA triplex formation. Since the length of the triplex structure can induce different clustering behaviors of bacterial cells, we therefore studied the effects of triplex structures of different lengths (10-bt, 12-bt, 16-bt, 20-bt, and 24-bt). Initially, two 5' terminal DBCO-modified strands (A and B) and two polypurine sequences containing strands (A* and B*) were premixed to form AA* and BB* via hybridization, respectively. The AA* modified *E. coli*-GFP and BB* modified *E. coli*-RFP were mixed in a 1:1 ratio (OD 600 = 0.5) with or without the strand C. After co-incubation for 5 h, 24-bt DNA triplex mediated the most prominent bacterial cell cluster under the confocal observation. Other groups also presented significant degrees of bacterial aggregation (Figure S26 A&B).

In order to perform quantitative analysis, the aggregation ratio can be statistically defined as a parameter to assess the bacterial assembly degree via DNA ternary interaction. The aggregation ratio was defined as the area occupied by bacterial clusters (whose area >15 μm^2) divided by the total area occupied by all bacteria. The average area of a single *E. coli* is 2.5 μm^2 thus only clusters of more than six bacterial cells are considered to be clusters. The quantitative analysis showed that in the presence of 24-nt strand C, 76% of the bacteria were integrated into clusters whereas only 21% of bacteria were clustered in the absence of strand C (Figure S26 C). Moreover, the average aggregated size of 24-nt group was 89 μm^2 under the interaction of triplex, which was significantly larger than that of the clusters observed in other groups (Figure S26 D).

Sequences (5' to 3')

Duplex design

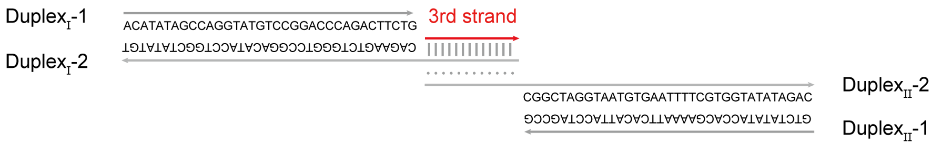

Figure S27. Sequence arrangement of duplexes.

Duplex$_{I}$-1	ACATATAGCCAGGTATGTCCGGACCCAGACTTCTG
Duplex$_{II}$-1	GTCTATATACCACGAAAATTCACATTACCTAGCCG
Duplex$_{I}$-2 (6-bt triplex)	GAAGAACAGAAGTCTGGGTCCGGACATACCTGGCTATATGT
Duplex$_{II}$-2 (6-bt triplex)	AAGAAGCGGCTAGGTAATGTGAATTTTCGTGGTATATAGAC
Duplex$_{I}$-2 (8-bt triplex)	GAAGAAGACAGAAGTCTGGGTCCGGACATACCTGGCTATATGT
Duplex$_{II}$-2 (8-bt triplex)	AGAAGAAGCGGCTAGGTAATGTGAATTTTCGTGGTATATAGAC
Duplex$_{I}$-2 (10-bt triplex)	GGGAAGAAGACAGAAGTCTGGGTCCGGACATACCTGGCTATATGT
Duplex$_{II}$-2 (10-bt triplex)	AGAAGAAGGGCGGCTAGGTAATGTGAATTTTCGTGGTATATAGAC
Duplex$_{I}$-2 (12-bt triplex)	AAGGGAAGAAGACAGAAGTCTGGGTCCGGACATACCTGGCTATATGT
Duplex$_{II}$-2 (12-bt triplex)	AGAAGAAGGGAACGGCTAGGTAATGTGAATTTTCGTGGTATATAGAC
Duplex$_{I}$-2 (14-bt triplex)	AAGGAGAGAAGGAGCAGAAGTCTGGGTCCGGACATACCTGGCTATATG T
Duplex$_{II}$-2 (14-bt triplex)	GAGGAAGAGAGGAACGGCTAGGTAATGTGAATTTTCGTGGTATATAGA C
strand C (6 nt)	TTCTTC
strand C (8 nt)	TCTTCTTC
strand C (10 nt)	TCTTCTTCCC
strand C (12 nt)	TCTTCTTCCCTT
strand C (14 nt)	CTCCTTCTCTCCTT

Table S1. Sequences of DNA duplexes. All sequences are written from 5' to 3'.

DX tile design

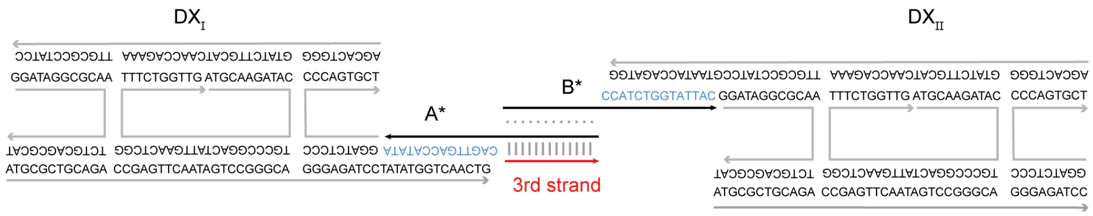

Figure S28. Sequence arrangement of DX tiles.

DX$_{I}$-1	AGCACTGGGGTATCTTGCATCAACCAGAAATTGCGCCTATCC
DX$_{I}$-2	GGATAGGCGCAATCTGCAGCGCAT
DX$_{I}$-3	ATGCAAGATACTGCCCGGACTATTGAACTCGGTTTCTGGTTG
DX$_{I}$-4	GGATCTCCCCCCAGTGCT
DX$_{I}$-5	ATGCGCTGCAGACCGAGTTCAATAGTCCGGGCAGGGAGATCC<br>TATATGGTCAACTG
DX$_{II}$-1	AGCACTGGGGTATCTTGCATCAACCAGAAATTGCGCCTATCCGTAATACCAG<br>ATGG
DX$_{II}$-2	GGATAGGCGCAATCTGCAGCGCAT
DX$_{II}$-3	ATGCAAGATACTGCCCGGACTATTGAACTCGGTTTCTGGTTG
DX$_{II}$-4	GGATCTCCCCCCAGTGCT
DX$_{II}$-5	ATGCGCTGCAGACCGAGTTCAATAGTCCGGGCAGGGAGATCC
A* (for 6-bt triplex)	GAAGAACAGTTGACCATATA
B* (for 6-bt triplex)	AAGAAGCCATCTGGTATTAC
A* (for 8-bt triplex)	GAAGAAGACAGTTGACCATATA
B* (for 8-bt triplex)	AGAAGAAGCCATCTGGTATTAC
A* (for 10-bt triplex)	GGGAAGAAGACAGTTGACCATATA
B* (for 10-bt triplex)	AGAAGAAGGGCCATCTGGTATTAC
A* (for 12-bt triplex)	AAGGGAAGAAGACAGTTGACCATATA
B* (for12-bt triplex)	AGAAGAAGGGAACCATCTGGTATTAC
A* (for 14-bt triplex)	AAGGAGAGAAGGAGCAGTTGACCATATA
B* (for 14-bt triplex)	GAGGAAGAGAGGAACCATCTGGTATTAC
strand C (6 nt)	TTCTTC
strand C (8 nt)	TCTTCTTC
strand C (10 nt)	TCTTCTTCCC
strand C (12 nt)	TCTTCTTCCCTT
strand C (14 nt)	CTCCTTCTCTCCTT

Table S2. Sequences of DNA DX tiles. All sequences are written from 5' to 3'.

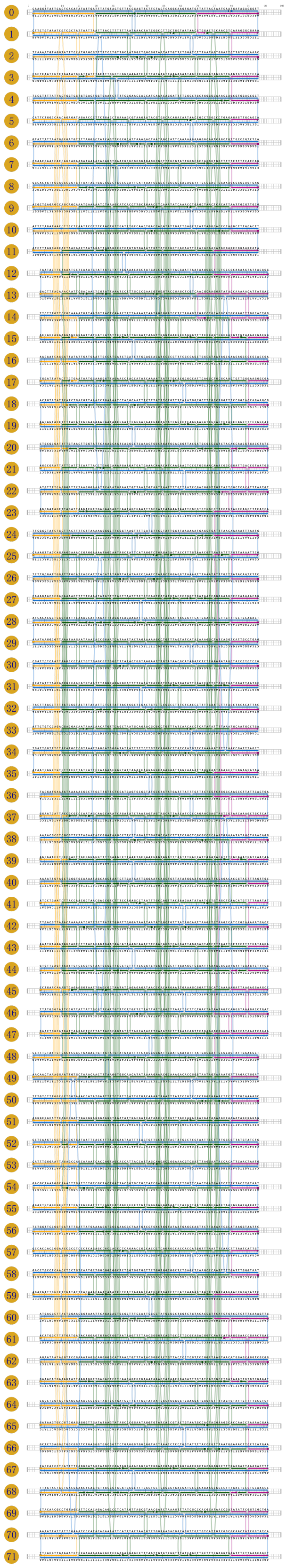

Figure S29. Cadnano design of cuboid origami structure.

Staple name	Sequence (5' to 3')
cuboid-core-1	CGGAAACAGTACATAAAATTTACCTTTTTTATGCA
cuboid-core-2	TGGTAATAACCATCACTTGCCTGAGTAGAAGAACT
cuboid-core-3	ACTACCCGTCGGATTAAAATTCGCGTCTGGCCAGC
cuboid-core-4	TCAATTGTTATGGTCATAGCTGTTTTGCCCGAACG
cuboid-core-5	GTGCCTAATGCACACAAGCCTTTATTAATTCGTAA
cuboid-core-6	AGAGCCGTCTGTGTGAAGTTGGCACCTTGCTAATG
cuboid-core-7	TTTAACTAACAACTAATAAGGAATTAATGAAAGTA
cuboid-core-8	TCATCCGCTCACAATTCAGCTCAATCAATATCTGG
cuboid-core-9	CAACTTATCATGAATTATTCATCAAATAATGGTTA
cuboid-core-10	ATTCATCCTACCAAGAAAACAAGCAAGCCGTCCAA
cuboid-core-11	TGGTGAACAAAGAGGCAAAGAATTAGCAAAAACAT
cuboid-core-12	TGAAAGTTTGAGCATCAAATCAACAGTTGAAGATT
cuboid-core-13	GGTCTGGCGACGCGCCATTCAGGCTGAGAGAAACC
cuboid-core-14	GTAGCCTCTTCTGTTGGTGCCGGAGCCAGCTCGTT
cuboid-core-15	TATTTGGTTTTTTTCCAGTCGGGAAAGCTAACCAG
cuboid-core-16	GGTTCAACGCTTTTAGGCAGTTGCTGGTTTTGCAT
cuboid-core-17	ATTAAATTCTATCACCATGATTCCCAATTATGAGC
cuboid-core-18	GCAAAGGGGGTTTGCGTGTGCCAGCTGCATTGAAC
cuboid-core-19	ACGTTCGGGAGTACCAAGTCAATTATCCCTTAAAG
cuboid-core-20	TGCTCGGGACGACGACAAAACTAGAAAAGCCCATA
cuboid-core-21	CAGGCGTAACCAACTCCGTGGGAACATTTAAAGAT
cuboid-core-22	CCAAACCAGGCCCTGAGCAGCTGATTGCCCTGCCA
cuboid-core-23	ACAGGAAAAATACATTGCTTTGAAAAACAATAAAT
cuboid-core-24	CCATATAAACTATCGGCCAATTACAAAACAAATTA
cuboid-core-25	CGCCTTATAATGCCAGTTTGAGGCGATGATGTTTA
cuboid-core-26	CATAAGATCGAATCGATCAAAGGCAGCTTAATTGC
cuboid-core-27	GAGCTATGTAAATATATTGCGCATTGAGAGAATAA
cuboid-core-28	ACAAGTGAATTTGCCATCAAAAATCAAAATTTTGC
cuboid-core-29	ACACCAACGTAGTCCACTATTAAACCGAGATGGCG
cuboid-core-30	ACGCTGAGAAGGCCCTTTAAATAGCAATAGCTATA
cuboid-core-31	AACTTACCGAAAGTCAATATTTCATTTGAATTCCT
cuboid-core-32	TCAAAACGTTATAGTCAGAAGCAGAAACAGTTCAG
cuboid-core-33	TGACAATAATTAACCCATATTTTGCATAACCCAGC
cuboid-core-34	ACTACCTTTGTTTTTTTAACCAATAGGAACATCAA
cuboid-core-35	ATTTAGCAAATAAACGGCGTAGATGAGGGTTGTGG
cuboid-core-36	ACTAAAAAGAACTGATTGGCTCGGCAAAATCCCAG
cuboid-core-37	GGTTTCAATCAACCAGACCGGAAAAAGAGAGAAAG
cuboid-core-38	TACAATCGTAGTATCGGAAACAGAGCACAGACAAT
cuboid-core-39	GTTTTTTAACCTCCGGCCATCAAGGTGCATCATCA
cuboid-core-40	AATCTTAATTCGAGTACCGGAATTATTTAGGACGG
cuboid-core-41	GTTTTTTTCAAATGCTGACCGAGGATTAAGAAAAG
cuboid-core-42	TTAACCGGAAAATAAGACGAGCGTAGTAGCGTTTC
cuboid-core-43	CTTCTGACCTAACGGATGTAGATTCTGAAAGGCGC

cuboid-core-44	AAAAATCCTGTTTGATGATACGTGAATAAAGCAAA
cuboid-core-45	AAGCAGAACCTCAGATGATGAACGCGCGGGGACTT
cuboid-core-46	TATAAGGCCGGGTAACGAGGATCCAAATATCAAAC
cuboid-core-47	GTTAGTTTCACATTAGATAAGAACGGTTTAAAGCC
cuboid-core-48	CTGTAAAGATGTCACGAATGCCTGTCACATTTGGG
cuboid-core-49	AATAACATGTATCCGGTCAAGCAAGCCGCCACAAA
cuboid-core-50	CAGACGCGCCGCATCAAAGTAGTAACCAGAACATG
cuboid-core-51	AAACATAAAGTCATGTAACAACAGTAATAAACATG
cuboid-core-52	ACCGGTGAGAGATATTCTCTATGCGTTATACATTT
cuboid-core-53	ATCCAAATCCTTCCAATAGATAATACATTTGACGA
cuboid-core-54	AGATAAGTCCGTACCGAGCTCGTCACTCTAGCCAG
cuboid-core-55	CCAGAAAAATATGCAGATCGAGCCAGTAATATTCC
cuboid-core-56	GCACGAGAAACAAATAACATCCCCAGAACGAGTAG
cuboid-core-57	GCGCTTTTATTCTTTCCTTATCATTATTTACGAAT
cuboid-core-58	TAGATTCTAATGCGGGAATTTTGCACGCTAAAACG
cuboid-core-59	TAAATCATACAGGCATACCGCACTATCTAATTTCT
cuboid-core-60	AAGAACTGCGAACGAGTAGTCAGGCACCCCTCTGCC
cuboid-core-61	CGCGAGCTGAGTTTGACTTCCATAAAGAATATAAT
cuboid-core-62	GACTGAACGCGCGACGACAGCATGTAACAATTGGA
cuboid-core-63	TTTTTCATTTTTTTAAATGAAAATTTTTAGATTAA
cuboid-core-64	AATGTACGGTAACATGTTGCGGATGCTCCTTTCAA
cuboid-core-65	TTTGCCCCAGCCGCCTGGCAAAGCGCCATTTACCT
cuboid-core-66	ATTAGAGGCATTTTCAATACCAGTTACTAGACAAT
cuboid-core-67	CTATTTATCCAAGCAAACTCCAACAGCAACATAGC
cuboid-core-68	GCTATCAGTTATAAAACCACTCATGTATCATCGCC
cuboid-core-69	ATTCTTTACAAGACGGGGGGTAATCAATAATGACT
cuboid-core-70	TTAATTTGCCATCGATATGGGAAGGATTATACCAA
cuboid-core-71	TATGATTAGAGAGCTTCTGAAAGCGGATTGCTGCC
cuboid-core-72	CAGAAACAGCTGTTTAGTATCATAGAACGGTCCCG
cuboid-core-73	AATTGGCTTAGTATCAGGCTATTTTTGCGCAACGC
cuboid-core-74	GAATTAGTTAATTTCTGGGGTTATATTCATAGGTC
cuboid-core-75	AGAAGAATTAGAAAACTGAAATACTTTTACACAGA
cuboid-core-76	CGCAAATTCAGGGAAGGCGTAACAGCAAGCCCAAT
cuboid-core-77	AGCATTAGCAAACCTCAAATGCTTTTTAAGCCGAG
cuboid-core-78	TAAAACGGAAAAGGAAATGCAAATATTCATTTTAC
cuboid-core-79	CCTTATTACGTACAACAGTTTCAGCGGAGTGTGAT
cuboid-core-80	AAATCGTCATGTAATAGATGACAACAACCTGTATC
cuboid-core-81	AATACATACACAGTATGAGAAACAATGAACGTGAG
cuboid-core-82	ATTCATTGAATCCCCGTAAAGAAATCCAGACTAAC
cuboid-core-83	ACAACTTTAATTTCTGTAGACAGCCCTCATACATG
cuboid-core-84	AACAGGCGAAGAATAGCGATCCAGAACAATATTAC
cuboid-core-85	AGAGACGATACTTTTGCGCTACAGAGGCTTTCATT
cuboid-core-86	TTTATCAAAATCGCGTTAGAAAAGACAAGAGCACT
cuboid-core-87	GACGGACGGAATAAGTTCAAGAATAAAGCGATATG

cuboid-core-88	GTCTAGCAGCTTTTGTTAAATAAGAGGGCTTCTTC
cuboid-core-89	AGGTCACCAGTCGCCACCATATAAGTATAGCCGGA
cuboid-core-90	AAATTATTCATTAACATACAATCCATCATAATATA
cuboid-core-91	AAGTAGTACCGGTGTATCACCGTACCCCAGCAAAA
cuboid-core-92	TTTCTTACCAACCCAGCACGCCAAACCGACAAGGA
cuboid-core-93	AATGCAAAATGAATTATTGAGGGATATGGTTCTAA
cuboid-core-94	GGTGATTAGCTGAGACTCCTCAAGGTTAATGACCC
cuboid-core-95	ATCTTATACCAGATTTAAAAGGTGTGTTTATCAAC
cuboid-core-96	TTAGAACCAATGAAACCAGTTACATAACAGTTCAA
cuboid-core-97	CTTTAGCCGCCCCCCCTGGTGTACTGGTAATAGCG
cuboid-core-98	TTAGCGTTTGCAAGCCTTAAGGCTTTCAGCTAAAT
cuboid-core-99	TCACAGAGCCATCAGATCATCGAGCGGGTATTAAA
cuboid-core-100	AAAGCATTGACCCTCAGTCATAATTCGGCATACAG
cuboid-core-101	TAACTCTGAATTTACCGTACAGGACCTATTTAAGA
cuboid-core-102	GCTGACCTTCATCAAGAAGCTCAGAGCCGCCGCAT
cuboid-core-103	TCTACGGTGTACAGACCTTGCGCAGACGGTCAATC
cuboid-core-104	GAACTGATAGACAGAGGTGGCAATCATCATAAGAG
cuboid-core-105	TAATCGTAAGAGTGGTTCCGAAAAGATTCACCTTCTGACTTCAGGTAAG
cuboid-core-106	AGTGAGGCGGATGTGCTGCAAGATACCATATAAAT
cuboid-core-107	CAGTATCCTCATTAAAGCCCACCACGAACCGCACA
cuboid-core-108	AAAAGTTTTAATAAACAAGCGCGAAACAAAGTACA
cuboid-core-109	CGTACGGGGTCAGTGCCAGGCGCATAGGCGAATGG
cuboid-core-110	GGAACGAGGAGAGATTTCTTTGACCTGTAATGCCA
cuboid-core-111	GGCGGGGTTTAGGGTTGCTCAGAAAGGGATACTGA
cuboid-core-112	ACGGTAACAGAGAGCCACCACCATTGGCTCATACG
cuboid-core-113	TGAGTTACTTTTTCAACAAAAATTGGGCTTGGTAA
cuboid-core-114	TTGAGAATACACCAACCAAGTTTCGAGGACTGGAG
cuboid-core-115	TTAGCATAGCCCCCTTGTGCAGCACTTACCATCTG
cuboid-core-116	GAACGGAATAGCCACCCAAACAGCATCGGAACGAG
cuboid-core-117	CTACGAAGGCACTAAAAGAACTAAGCGATTTTACA
cuboid-core-118	GTAAAATACCAAGCAACGGGGATCGTTATCGCCCA
cuboid-core-119	AAAATACCACAAGATTCGTAGCTCGTCTGGACTAG
cuboid-core-120	AGGTCAGAACAGCACCACGTAATCCTTTCCACAAG
cuboid-core-121	GGTGGAGGTTGCCGGAAACGTCGACGAGGCAACCA
cuboid-core-122	GTTATTCCACATGGGATCCAAAAAAAAGGCTCCAA
cuboid-core-123	TACTAAATATTGACGATTCACAATACATATAAGAA
cuboid-core-124	AGCGAAAGCCGTTAGCGGTTAGTAGAGCCTTTAAT
cuboid-core-125	GATCTAAAGTTCACCCTCTCGTTTTAGTAAGAGGT
cuboid-core-126	CGCATAACCGAAAGGCCGAAAACCAGCCAAAATTT
cuboid-core-127	GGGACGCCAGTTTCCCATCAAAAGTCATATAGGGG
cuboid-core-128	AAGAATGAATGGTGGCACAATAGATAATATCCAGG
cuboid-core-129	TGTATCGGTTTCGTCTTCGCAAAGACACCGGAAAT
cuboid-core-130	AGCTTGCTTTCGAGGTGGCCGACATAAAATGGAAG
cuboid-core-131	TTAAAGATAAACCCTAAAAGAACCCAGTCACAGAT

cuboid-core-132	AATCGGCCAGGCGGTCAAATCTAAAGTAACATCGT
cuboid-core-133	CTCCCTATTAATTTTAATTGCGATTAAGTTGGAGA
cuboid-core-134	CTGACCTGTCATTGGGCTCTTTTTGAATGGCTATT

Table S3. Sequences of core staples of DNA origami cuboid. All sequences are written from 5' to 3'.

cuboid-right-135	ttttAACCGTCTATCATTGATTAGTAATAAGTGG
cuboid-right-136	ttttAAGTGTAAAGCCAATTGCGTTGCGtttt
cuboid-right-137	ttttCCTGTAATACTTTTGCCCAAAAAAGCTTGCCGTT
cuboid-right-138	GTCGAACGCAAGGATATAGGGAGAACATACGAGCCGGAAGCATAtttt
cuboid-right-139	ttttCCAGTGCCACATTATGACtttt
cuboid-right-140	ttttTCGGTGCGGAAACGACGGtttt
cuboid-right-141	ttttCGCTTCTGGGAAGGGCGAtttt
cuboid-right-142	ttttGTTTGGAACAAGCAAAGGGCGAAAtttt
cuboid-right-143	GGTGGATTGAGTGAGCGTAGCCAGCTTTCATCAtttt
cuboid-right-144	ttttGATAGGTCATTCCGGCACtttt
cuboid-right-145	ttttACATTAAATCCGTAATGGtttt
cuboid-right-146	ttttCATTAAATTTTTGTTAAATCTTCCTGAGTA
cuboid-right-147	ttttGAAGATTGTATATGTTAAAATTCGtttt
cuboid-right-148	ttttAGTCTGGAGCAACCCCAAAAACAGtttt
cuboid-right-149	ttttCGGAGAGGGTAGTCATTGCCTGAGtttt
cuboid-right-150	ttttGTAATGTGTAGGATAAATTAATGCtttt
cuboid-right-151	ttttAGCTAAATCGGTTGCAATGCCTGAtttt
cuboid-right-152	ttttTCAGTGAATAAGGCTTTAACAAACTATATTCGCA
cuboid-right-153	TCGGCCCTGAATAAAGCCTCAGAGCATAAtttt
cuboid-right-154	ttttCCTGTTTAGGCTGCTCATtttt
cuboid-right-155	ttttAGCGGTCCACGCAGTGTTGTTCCAtttt
cuboid-right-156	ttttGCAACTAAAGGTCAATAAtttt
cuboid-right-157	ttttGGTCATTTTTTTAAATATtttt
cuboid-right-158	ATAAGATTAATTACCCTTGACCATAAATCAAAAtttt
cuboid-right-159	ttttCGAAAGACTTTGATAAGAtttt
cuboid-right-160	ttttATCAGGTCTGAGGAAGCCtttt
cuboid-right-161	ttttAGCGTCCAATACTGCGGAAACGAGAAGACTATTAAT
cuboid-right-162	ttttGCTTTTGCAAAATTTAGACTGGATtttt
cuboid-right-163	ttttCAGATACATAACAAATAGCGAGAGtttt
cuboid-right-164	ttttATTACAGGTAGAATTCAACTAATGtttt
cuboid-right-165	ttttGAATTACCTTATCGGAACAACATTtttt
cuboid-right-166	ttttATTACCCAAATCTTTAATCATTGTtttt
cuboid-right-167	ttttTGAGACGGGCAAAGAGTTGCAGCAtttt
cuboid-right-168	ttttCAACTTTGAAAGAGGAAGGGAACGCTCCATTAAA
cuboid-right-169	ATACAGATGATGACAAGAACCGGATATTCtttt
cuboid-right-170	ttttCCGCGACCTCGAACTGACtttt
cuboid-right-171	ttttAGAGGCAAATGTCGAAATtttt
cuboid-right-172	ttttTTCATGAGGTAAAACGAAtttt
cuboid-right-173	TTATATATTCTAGTTGCAATTTCTTAAACAGCTtttt
cuboid-right-174	ttttGGCTTGCAGAAAGACTTTtttt
cuboid-right-175	ttttTGATACCGAGGTCGCTGAtttt
cuboid-right-176	ttttCTCACTGCCCGCTCTTTTCACCAGtttt
cuboid-left-177	ttttCTTCTGTAAATCTGAAAACATtttt
cuboid-left-178	ttttATCTTTAGGAGCGAAGTATTAtttt

cuboid-left-179	ttttGACTTTACAAGAAACCAATCAtttt
cuboid-left-180	GCGTTTGCGGAGCAGCAGAGGAAGGTTATCTAAAATtttt
cuboid-left-181	ttttACCACCAGAAAAGGTAAAGTAtttt
cuboid-left-182	TGATATAATCCAGCAGACACCGCCTGCAACAGTGCCtttt
cuboid-left-183	ttttTGGATTATAAATTGAGAATCGtttt
cuboid-left-184	TGACAGAGATACATCGCCATTAAAAATACtttt
cuboid-left-185	ttttAACAGTACCCGACCGTGTGATtttt
cuboid-left-186	AAATGAAATGCGACCAGTAATAAAAGGGAtttt
cuboid-left-187	ttttAGGCGAATTCCAATCGCAAGAtttt
cuboid-left-188	ttttAGCGATAGCCAGATAGCCGAAtttt
cuboid-left-189	TAAGTTAGATTGAATCCTGTCGCTAGGAAATACCTACATTTTGAtttt
cuboid-left-190	ttttCAAAGTTACCAGTACCCAAAAtttt
cuboid-left-191	ttttCAAAGAACGCGAACTGAACACtttt
cuboid-left-192	ttttAAATAAGGCGTTTAACGTCAAtttt
cuboid-left-193	ttttCGCTCAATCGTCATCGCGCAGtttt
cuboid-left-194	TATTCGCTCATTTAATTATCAATATATGTGAGTGAATAACCTTGtttt
cuboid-left-195	ttttCCATATTTAACATACAATTTTtttt
cuboid-left-196	ttttATTCTGTCCAGAAGGCGTTTTtttt
cuboid-left-197	ttttATAATCGGCTGTTTCATCGTAtttt
cuboid-left-198	ttttGGAATCATTTTGAGGCAGGTCtttt
cuboid-left-199	ttttAGCGAACCTACCGGAACCAGAtttt
cuboid-left-200	ttttATCCTGAATGCCTTTAGCGTCtttt
cuboid-left-201	ttttAAATGAAAAACCGACTTGAGCtttt
cuboid-left-202	ttttCCTGAACAAGCCAAAGACAAAtttt
cuboid-left-203	ttttGAACTGGCAAGAATAGAAAGGtttt
cuboid-left-204	ttttAACAACTAAAGGAATTGTGTACCAGCAGTC
cuboid-left-205	ttttCATTCTGGCCAAATATACAGTtttt
cuboid-left-206	ttttAGGGCGACATTCCAGTACAAAtttt
cuboid-left-207	ttttCATTTGGGAATTCCTCAGAGCtttt
cuboid-left-208	ttttAGACTGTAGCGCTACCAGGCGtttt
cuboid-left-209	ttttGCCACCACCGGATATTATTCTtttt
cuboid-left-210	ttttAGACGATTGGCCAAGCGTCATtttt
cuboid-left-211	ttttACATGGCTTTTGATGATTCCAGTTTGATATTCAC
cuboid-left-212	ttttGAAACATGAAAGTATTCGGAACCACCGCCTCAGGAGGACC
cuboid-left-213	ttttGATAAGTGCCGTCGAGTGCTCAGGTTTTCACAAAATCCCC
cuboid-left-214	ttttCACCACCCTCATTTTCCCGCCACAGAGCCACAAG
cuboid-left-215	ttttCTACAACGCCTGTAGCTCGTCACAACCGATCACC
cuboid-left-216	ttttCGAACGAACCACCTGATTGTTtttt
cuboid-left-217	ttttTTCACGTTGAAAATCTTTCGAATAATAATTTTtttt
cuboid-left-218	ttttACGCTGAGAGCCAACAAAGAAtttt

Table S4. Sequences of edge staples of DNA origami cuboid (valency number of 0). All sequences are written from 5' to 3'.

cuboid-right-135	ttttAACCGTCTATCATTGATTAGTAATAAGTGG
cuboid-right-136	ttttAAGTGTAAAGCCAATTGCGTTGCGtttt
cuboid-right-137	ttttCCTGTAATACTTTTGCCCAAAAAAGCTTGCCGTT
cuboid-right-138	GTCGAACGCAAGGATATAGGGAGAACATACGAGCCGGAAGCATAtttt
cuboid-right-139	ttttCCAGTGCCACATTATGACtttt
cuboid-right-140	ttttTCGGTGCGGAAACGACGGtttt
cuboid-right-141	ttttCGCTTCTGGGAAGGGCGAtttt
cuboid-right-142	ttttGTTTGGAACAAGCAAAGGGCGAAAtttt
cuboid-right-143	GGTGGATTGAGTGAGCGTAGCCAGCTTTCATCAtttt
cuboid-right-144	ttttGATAGGTCATTCCGGCACtttt
cuboid-right-145	ttttACATTAAATCCGTAATGGtttt
cuboid-right-146	ttttCATTAAATTTTTGTTAAATCTTCCTGAGTA
cuboid-right-147	ttttGAAGATTGTATATGTTAAAATTCGtttt
cuboid-right-148	ttttAGTCTGGAGCAACCCCAAAAACAGtttt
cuboid-right-149	ttttCGGAGAGGGTAGTCATTGCCTGAGGTAATACCAGATGG
cuboid-right-150	ttttGTAATGTGTAGGATAAATTAATGCtttt
cuboid-right-151	ttttAGCTAAATCGGTTGCAATGCCTGAtttt
cuboid-right-152	ttttTCAGTGAATAAGGCTTTAACAAACTATATTCGCA
cuboid-right-153	TCGGCCCTGAATAAAGCCTCAGAGCATAAtttt
cuboid-right-154	ttttCCTGTTTAGGCTGCTCATtttt
cuboid-right-155	ttttAGCGGTCCACGCAGTGTTGTTCCAtttt
cuboid-right-156	ttttGCAACTAAAGGTCAATAAtttt
cuboid-right-157	ttttGGTCATTTTTTTAAATATtttt
cuboid-right-158	ATAAGATTAATTACCCTTGACCATAAATCAAAAtttt
cuboid-right-159	ttttCGAAAGACTTTGATAAGAtttt
cuboid-right-160	ttttATCAGGTCTGAGGAAGCCtttt
cuboid-right-161	ttttAGCGTCCAATACTGCGGAAACGAGAAGACTATTAAT
cuboid-right-162	ttttGCTTTTGCAAAATTTAGACTGGATtttt
cuboid-right-163	ttttCAGATACATAACAAATAGCGAGAGtttt
cuboid-right-164	ttttATTACAGGTAGAATTCAACTAATGtttt
cuboid-right-165	ttttGAATTACCTTATCGGAACAACATTtttt
cuboid-right-166	ttttATTACCCAAATCTTTAATCATTGTtttt
cuboid-right-167	ttttTGAGACGGGCAAAGAGTTGCAGCAtttt
cuboid-right-168	ttttCAACTTTGAAAGAGGAAGGGAACGCTCCATTAAA
cuboid-right-169	ATACAGATGATGACAAGAACCGGATATTCtttt
cuboid-right-170	ttttCCGCGACCTCGAACTGACtttt
cuboid-right-171	ttttAGAGGCAAATGTCGAAATtttt
cuboid-right-172	ttttTTCATGAGGTAAAACGAAtttt
cuboid-right-173	TTATATATTCTAGTTGCAATTTCTTAAACAGCTtttt
cuboid-right-174	ttttGGCTTGCAGAAAGACTTTtttt
cuboid-right-175	ttttTGATACCGAGGTCGCTGAtttt
cuboid-right-176	ttttCTCACTGCCCGCTCTTTTCACCAGtttt
cuboid-left-177	ttttCTTCTGTAAATCTGAAAACATtttt
cuboid-left-178	ttttATCTTTAGGAGCGAAGTATTAtttt

cuboid-left-179	ttttGACTTTACAAGAAACCAATCAtttt
cuboid-left-180	GCGTTTGCGGAGCAGCAGAGGAAGGTTATCTAAAATtttt
cuboid-left-181	ttttACCACCAGAAAAGGTAAAGTAtttt
cuboid-left-182	TGATATAATCCAGCAGACACCGCCTGCAACAGTGCCtttt
cuboid-left-183	ttttTGGATTATAAATTGAGAATCGtttt
cuboid-left-184	TGACAGAGATACATCGCCATTAAAAATACtttt
cuboid-left-185	ttttAACAGTACCCGACCGTGTGATtttt
cuboid-left-186	AAATGAAATGCGACCAGTAATAAAAGGGAtttt
cuboid-left-187	ttttAGGCGAATTCCAATCGCAAGAtttt
cuboid-left-188	ttttAGCGATAGCCAGATAGCCGAAtttt
cuboid-left-189	TAAGTTAGATTGAATCCTGTCGCTAGGAAATACCTACATTTTGAtttt
cuboid-left-190	ttttCAAAGTTACCAGTACCCAAAAtttt
cuboid-left-191	ttttCAAAGAACGCGAACTGAACACtttt
cuboid-left-192	ttttAAATAAGGCGTTTAACGTCAATATATGGTCAACTG
cuboid-left-193	ttttCGCTCAATCGTCATCGCGCAGtttt
cuboid-left-194	TATTCGCTCATTTAATTATCAATATATGTGAGTGAATAACCTTGtttt
cuboid-left-195	ttttCCATATTTAACATACAATTTTtttt
cuboid-left-196	ttttATTCTGTCCAGAAGGCGTTTTtttt
cuboid-left-197	ttttATAATCGGCTGTTTCATCGTAtttt
cuboid-left-198	ttttGGAATCATTTTGAGGCAGGTCtttt
cuboid-left-199	ttttAGCGAACCTACCGGAACCAGAtttt
cuboid-left-200	ttttATCCTGAATGCCTTTAGCGTCtttt
cuboid-left-201	ttttAAATGAAAAACCGACTTGAGCtttt
cuboid-left-202	ttttCCTGAACAAGCCAAAGACAAAtttt
cuboid-left-203	ttttGAACTGGCAAGAATAGAAAGGtttt
cuboid-left-204	ttttAACAACTAAAGGAATTGTGTACCAGCAGTC
cuboid-left-205	ttttCATTCTGGCCAAATATACAGTtttt
cuboid-left-206	ttttAGGGCGACATTCCAGTACAAAtttt
cuboid-left-207	ttttCATTTGGGAATTCCTCAGAGCtttt
cuboid-left-208	ttttAGACTGTAGCGCTACCAGGCGtttt
cuboid-left-209	ttttGCCACCACCGGATATTATTCTtttt
cuboid-left-210	ttttAGACGATTGGCCAAGCGTCATtttt
cuboid-left-211	ttttACATGGCTTTTGATGATTCCAGTTTGATATTCAC
cuboid-left-212	ttttGAAACATGAAAGTATTCGGAACCACCGCCTCAGGAGGACC
cuboid-left-213	ttttGATAAGTGCCGTCGAGTGCTCAGGTTTTCACAAAATCCCC
cuboid-left-214	ttttCACCACCCTCATTTTCCCGCCACAGAGCCACAAG
cuboid-left-215	ttttCTACAACGCCTGTAGCTCGTCACAACCGATCACC
cuboid-left-216	ttttCGAACGAACCACCTGATTGTTtttt
cuboid-left-217	ttttTTCACGTTGAAAATCTTTCGAATAATAATTTTtttt
cuboid-left-218	ttttACGCTGAGAGCCAACAAAGAAtttt

Table S5. Sequences of edge staples of DNA origami cuboid (valency number of 1). All sequences are written from 5' to 3'.

cuboid-right-135	ttttAACCGTCTATCATTGATTAGTAATAAGTGG
cuboid-right-136	ttttAAGTGTAAAGCCAATTGCGTTGCGtttt
cuboid-right-137	ttttCCTGTAATACTTTTGCCCAAAAAAGCTTGCCGTT
cuboid-right-138	GTCGAACGCAAGGATATAGGGAGAACATACGAGCCGGAAGCATAtttt
cuboid-right-139	ttttCCAGTGCCACATTATGACtttt
cuboid-right-140	ttttTCGGTGCGGAAACGACGGtttt
cuboid-right-141	ttttCGCTTCTGGGAAGGGCGAtttt
cuboid-right-142	ttttGTTTGGAACAAGCAAAGGGCGAAAtttt
cuboid-right-143	GGTGGATTGAGTGAGCGTAGCCAGCTTTCATCAtttt
cuboid-right-144	ttttGATAGGTCATTCCGGCACtttt
cuboid-right-145	ttttACATTAAATCCGTAATGGtttt
cuboid-right-146	ttttCATTAAATTTTTGTTAAATCTTCCTGAGTA
cuboid-right-147	ttttGAAGATTGTATATGTTAAAATTCGtttt
cuboid-right-148	ttttAGTCTGGAGCAACCCCAAAAACAGtttt
cuboid-right-149	ttttCGGAGAGGGTAGTCATTGCCTGAGGTAATACCAGATGG
cuboid-right-150	ttttGTAATGTGTAGGATAAATTAATGCtttt
cuboid-right-151	ttttAGCTAAATCGGTTGCAATGCCTGAtttt
cuboid-right-152	ttttTCAGTGAATAAGGCTTTAACAAACTATATTCGCA
cuboid-right-153	TCGGCCCTGAATAAAGCCTCAGAGCATAAtttt
cuboid-right-154	ttttCCTGTTTAGGCTGCTCATtttt
cuboid-right-155	ttttAGCGGTCCACGCAGTGTTGTTCCAtttt
cuboid-right-156	ttttGCAACTAAAGGTCAATAAtttt
cuboid-right-157	ttttGGTCATTTTTTTAAATATtttt
cuboid-right-158	ATAAGATTAATTACCCTTGACCATAAATCAAAAtttt
cuboid-right-159	ttttCGAAAGACTTTGATAAGAtttt
cuboid-right-160	ttttATCAGGTCTGAGGAAGCCtttt
cuboid-right-161	ttttAGCGTCCAATACTGCGGAAACGAGAAGACTATTAAT
cuboid-right-162	ttttGCTTTTGCAAAATTTAGACTGGATtttt
cuboid-right-163	ttttCAGATACATAACAAATAGCGAGAGtttt
cuboid-right-164	ttttATTACAGGTAGAATTCAACTAATGGTAATACCAGATGG
cuboid-right-165	ttttGAATTACCTTATCGGAACAACATTtttt
cuboid-right-166	ttttATTACCCAAATCTTTAATCATTGTtttt
cuboid-right-167	ttttTGAGACGGGCAAAGAGTTGCAGCAGTAATACCAGATGG
cuboid-right-168	ttttCAACTTTGAAAGAGGAAGGGAACGCTCCATTAAA
cuboid-right-169	ATACAGATGATGACAAGAACCGGATATTCtttt
cuboid-right-170	ttttCCGCGACCTCGAACTGACtttt
cuboid-right-171	ttttAGAGGCAAATGTCGAAATtttt
cuboid-right-172	ttttTTCATGAGGTAAAACGAAtttt
cuboid-right-173	TTATATATTCTAGTTGCAATTTCTTAAACAGCTtttt
cuboid-right-174	ttttGGCTTGCAGAAAGACTTTtttt
cuboid-right-175	ttttTGATACCGAGGTCGCTGAtttt
cuboid-right-176	ttttCTCACTGCCCGCTCTTTTCACCAGtttt
cuboid-left-177	ttttCTTCTGTAAATCTGAAAACATtttt
cuboid-left-178	ttttATCTTTAGGAGCGAAGTATTAtttt

cuboid-left-179	ttttGACTTTACAAGAAACCAATCAtttt
cuboid-left-180	GCGTTTGCGGAGCAGCAGAGGAAGGTTATCTAAAATtttt
cuboid-left-181	ttttACCACCAGAAAAGGTAAAGTAtttt
cuboid-left-182	TGATATAATCCAGCAGACACCGCCTGCAACAGTGCCtttt
cuboid-left-183	ttttTGGATTATAAATTGAGAATCGtttt
cuboid-left-184	TGACAGAGATACATCGCCATTAAAAATACtttt
cuboid-left-185	ttttAACAGTACCCGACCGTGTGATtttt
cuboid-left-186	AAATGAAATGCGACCAGTAATAAAAGGGAtttt
cuboid-left-187	ttttAGGCGAATTCCAATCGCAAGAtttt
cuboid-left-188	ttttAGCGATAGCCAGATAGCCGAAtttt
cuboid-left-189	TAAGTTAGATTGAATCCTGTCGCTAGGAAATACCTACATTTTGAtttt
cuboid-left-190	ttttCAAAGTTACCAGTACCCAAAAtttt
cuboid-left-191	ttttCAAAGAACGCGAACTGAACACtttt
cuboid-left-192	ttttAAATAAGGCGTTTAACGTCAATATATGGTCAACTG
cuboid-left-193	ttttCGCTCAATCGTCATCGCGCAGtttt
cuboid-left-194	TATTCGCTCATTTAATTATCAATATATGTGAGTGAATAACCTTGtttt
cuboid-left-195	ttttCCATATTTAACATACAATTTTtttt
cuboid-left-196	ttttATTCTGTCCAGAAGGCGTTTTtttt
cuboid-left-197	ttttATAATCGGCTGTTTCATCGTAtttt
cuboid-left-198	ttttGGAATCATTTTGAGGCAGGTCtttt
cuboid-left-199	ttttAGCGAACCTACCGGAACCAGAtttt
cuboid-left-200	ttttATCCTGAATGCCTTTAGCGTCtttt
cuboid-left-201	ttttAAATGAAAAACCGACTTGAGCtttt
cuboid-left-202	ttttCCTGAACAAGCCAAAGACAAAtttt
cuboid-left-203	ttttGAACTGGCAAGAATAGAAAGGtttt
cuboid-left-204	ttttAACAACTAAAGGAATTGTGTACCAGCAGTC
cuboid-left-205	ttttCATTCTGGCCAAATATACAGTTATATGGTCAACTG
cuboid-left-206	ttttAGGGCGACATTCCAGTACAAAtttt
cuboid-left-207	ttttCATTTGGGAATTCCTCAGAGCTATATGGTCAACTG
cuboid-left-208	ttttAGACTGTAGCGCTACCAGGCGtttt
cuboid-left-209	ttttGCCACCACCGGATATTATTCTtttt
cuboid-left-210	ttttAGACGATTGGCCAAGCGTCATtttt
cuboid-left-211	ttttACATGGCTTTTGATGATTCCAGTTTGATATTCAC
cuboid-left-212	ttttGAAACATGAAAGTATTCGGAACCACCGCCTCAGGAGGACC
cuboid-left-213	ttttGATAAGTGCCGTCGAGTGCTCAGGTTTTCACAAAATCCCC
cuboid-left-214	ttttCACCACCCTCATTTTCCCGCCACAGAGCCACAAG
cuboid-left-215	ttttCTACAACGCCTGTAGCTCGTCACAACCGATCACC
cuboid-left-216	ttttCGAACGAACCACCTGATTGTTtttt
cuboid-left-217	ttttTTCACGTTGAAAATCTTTCGAATAATAATTTTtttt
cuboid-left-218	ttttACGCTGAGAGCCAACAAAGAAtttt

Table S6. Sequences of edge staples of DNA origami cuboid (valency number of 3). All sequences are written from 5' to 3'.

cuboid-right-135	ttttAACCGTCTATCATTGATTAGTAATAAGTGG
cuboid-right-136	ttttAAGTGTAAAGCCAATTGCGTTGCGtttt
cuboid-right-137	ttttCCTGTAATACTTTTGCCCAAAAAAGCTTGCCGTT
cuboid-right-138	GTCGAACGCAAGGATATAGGGAGAACATACGAGCCGGAAGCATAtttt
cuboid-right-139	ttttCCAGTGCCACATTATGACtttt
cuboid-right-140	ttttTCGGTGCGGAAACGACGGtttt
cuboid-right-141	ttttCGCTTCTGGGAAGGGCGAtttt
cuboid-right-142	ttttGTTTGGAACAAGCAAAGGGCGAAAtttt
cuboid-right-143	GGTGGATTGAGTGAGCGTAGCCAGCTTTCATCAtttt
cuboid-right-144	ttttGATAGGTCATTCCGGCACtttt
cuboid-right-145	ttttACATTAAATCCGTAATGGtttt
cuboid-right-146	ttttCATTAAATTTTTGTTAAATCTTCCTGAGTA
cuboid-right-147	ttttGAAGATTGTATATGTTAAAATTCGtttt
cuboid-right-148	ttttAGTCTGGAGCAACCCCAAAAACAGGTAATACCAGATGG
cuboid-right-149	ttttCGGAGAGGGTAGTCATTGCCTGAGtttt
cuboid-right-150	ttttGTAATGTGTAGGATAAATTAATGCGTAATACCAGATGG
cuboid-right-151	ttttAGCTAAATCGGTTGCAATGCCTGAtttt
cuboid-right-152	ttttTCAGTGAATAAGGCTTTAACAAACTATATTCGCA
cuboid-right-153	TCGGCCCTGAATAAAGCCTCAGAGCATAAtttt
cuboid-right-154	ttttCCTGTTTAGGCTGCTCATtttt
cuboid-right-155	ttttAGCGGTCCACGCAGTGTTGTTCCAGTAATACCAGATGG
cuboid-right-156	ttttGCAACTAAAGGTCAATAAtttt
cuboid-right-157	ttttGGTCATTTTTTTAAATATtttt
cuboid-right-158	ATAAGATTAATTACCCTTGACCATAAATCAAAAtttt
cuboid-right-159	ttttCGAAAGACTTTGATAAGAtttt
cuboid-right-160	ttttATCAGGTCTGAGGAAGCCtttt
cuboid-right-161	ttttAGCGTCCAATACTGCGGAAACGAGAAGACTATTAAT
cuboid-right-162	ttttGCTTTTGCAAAATTTAGACTGGATtttt
cuboid-right-163	ttttCAGATACATAACAAATAGCGAGAGGTAATACCAGATGG
cuboid-right-164	ttttATTACAGGTAGAATTCAACTAATGtttt
cuboid-right-165	ttttGAATTACCTTATCGGAACAACATTGTAATACCAGATGG
cuboid-right-166	ttttATTACCCAAATCTTTAATCATTGTtttt
cuboid-right-167	ttttTGAGACGGGCAAAGAGTTGCAGCAtttt
cuboid-right-168	ttttCAACTTTGAAAGAGGAAGGGAACGCTCCATTAAA
cuboid-right-169	ATACAGATGATGACAAGAACCGGATATTCtttt
cuboid-right-170	ttttCCGCGACCTCGAACTGACtttt
cuboid-right-171	ttttAGAGGCAAATGTCGAAATtttt
cuboid-right-172	ttttTTCATGAGGTAAAACGAAtttt
cuboid-right-173	TTATATATTCTAGTTGCAATTTCTTAAACAGCTtttt
cuboid-right-174	ttttGGCTTGCAGAAAGACTTTtttt
cuboid-right-175	ttttTGATACCGAGGTCGCTGAtttt
cuboid-right-176	ttttCTCACTGCCCGCTCTTTTCACCAGGTAATACCAGATGG
cuboid-left-177	ttttCTTCTGTAAATCTGAAAACATtttt
cuboid-left-178	ttttATCTTTAGGAGCGAAGTATTAtttt

cuboid-left-179	ttttGACTTTACAAGAAACCAATCAtttt
cuboid-left-180	GCGTTTGCGGAGCAGCAGAGGAAGGTTATCTAAAATtttt
cuboid-left-181	ttttACCACCAGAAAAGGTAAAGTAtttt
cuboid-left-182	TGATATAATCCAGCAGACACCGCCTGCAACAGTGCCtttt
cuboid-left-183	ttttTGGATTATAAATTGAGAATCGtttt
cuboid-left-184	TGACAGAGATACATCGCCATTAAAAATACtttt
cuboid-left-185	ttttAACAGTACCCGACCGTGTGATtttt
cuboid-left-186	AAATGAAATGCGACCAGTAATAAAAGGGAtttt
cuboid-left-187	ttttAGGCGAATTCCAATCGCAAGAtttt
cuboid-left-188	ttttAGCGATAGCCAGATAGCCGAAtttt
cuboid-left-189	TAAGTTAGATTGAATCCTGTCGCTAGGAAATACCTACATTTTGAtttt
cuboid-left-190	ttttCAAAGTTACCAGTACCCAAAAtttt
cuboid-left-191	ttttCAAAGAACGCGAACTGAACACTATATGGTCAACTG
cuboid-left-192	ttttAAATAAGGCGTTTAACGTCAAtttt
cuboid-left-193	ttttCGCTCAATCGTCATCGCGCAGTATATGGTCAACTG
cuboid-left-194	TATTCGCTCATTTAATTATCAATATATGTGAGTGAATAACCTTGtttt
cuboid-left-195	ttttCCATATTTAACATACAATTTTTATATGGTCAACTG
cuboid-left-196	ttttATTCTGTCCAGAAGGCGTTTTtttt
cuboid-left-197	ttttATAATCGGCTGTTTCATCGTAtttt
cuboid-left-198	ttttGGAATCATTTTGAGGCAGGTCtttt
cuboid-left-199	ttttAGCGAACCTACCGGAACCAGAtttt
cuboid-left-200	ttttATCCTGAATGCCTTTAGCGTCtttt
cuboid-left-201	ttttAAATGAAAAACCGACTTGAGCtttt
cuboid-left-202	ttttCCTGAACAAGCCAAAGACAAAtttt
cuboid-left-203	ttttGAACTGGCAAGAATAGAAAGGtttt
cuboid-left-204	ttttAACAACTAAAGGAATTGTGTACCAGCAGTC
cuboid-left-205	ttttCATTCTGGCCAAATATACAGTtttt
cuboid-left-206	ttttAGGGCGACATTCCAGTACAAATATATGGTCAACTG
cuboid-left-207	ttttCATTTGGGAATTCCTCAGAGCtttt
cuboid-left-208	ttttAGACTGTAGCGCTACCAGGCGTATATGGTCAACTG
cuboid-left-209	ttttGCCACCACCGGATATTATTCTtttt
cuboid-left-210	ttttAGACGATTGGCCAAGCGTCATtttt
cuboid-left-211	ttttACATGGCTTTTGATGATTCCAGTTTGATATTCAC
cuboid-left-212	ttttGAAACATGAAAGTATTCGGAACCACCGCCTCAGGAGGACC
cuboid-left-213	ttttGATAAGTGCCGTCGAGTGCTCAGGTTTTCACAAAATCCCC
cuboid-left-214	ttttCACCACCCTCATTTTCCCGCCACAGAGCCACAAG
cuboid-left-215	ttttCTACAACGCCTGTAGCTCGTCACAACCGATCACC
cuboid-left-216	ttttCGAACGAACCACCTGATTGTTTATATGGTCAACTG
cuboid-left-217	ttttTTCACGTTGAAAATCTTTCGAATAATAATTTTtttt
cuboid-left-218	ttttACGCTGAGAGCCAACAAAGAAtttt

Table S7. Sequences of edge staples of DNA origami cuboid (valency number of 6). All sequences are written from 5' to 3'.

cuboid-right-135	ttttAACCGTCTATCATTGATTAGTAATAAGTGG
cuboid-right-136	ttttAAGTGTAAAGCCAATTGCGTTGCGGTAATACCAGATGG
cuboid-right-137	ttttCCTGTAATACTTTTGCCCAAAAAAGCTTGCCGTT
cuboid-right-138	GTCGAACGCAAGGATATAGGGAGAACATACGAGCCGGAAGCATAtttt
cuboid-right-139	ttttCCAGTGCCACATTATGACtttt
cuboid-right-140	ttttTCGGTGCGGAAACGACGGtttt
cuboid-right-141	ttttCGCTTCTGGGAAGGGCGAtttt
cuboid-right-142	ttttGTTTGGAACAAGCAAAGGGCGAAAGTAATACCAGATGG
cuboid-right-143	GGTGGATTGAGTGAGCGTAGCCAGCTTTCATCAtttt
cuboid-right-144	ttttGATAGGTCATTCCGGCACtttt
cuboid-right-145	ttttACATTAAATCCGTAATGGtttt
cuboid-right-146	ttttCATTAAATTTTTGTTAAATCTTCCTGAGTA
cuboid-right-147	ttttGAAGATTGTATATGTTAAAATTCGGTAATACCAGATGG
cuboid-right-148	ttttAGTCTGGAGCAACCCCAAAAACAGtttt
cuboid-right-149	ttttCGGAGAGGGTAGTCATTGCCTGAGGTAATACCAGATGG
cuboid-right-150	ttttGTAATGTGTAGGATAAATTAATGCtttt
cuboid-right-151	ttttAGCTAAATCGGTTGCAATGCCTGAGTAATACCAGATGG
cuboid-right-152	ttttTCAGTGAATAAGGCTTTAACAAACTATATTCGCA
cuboid-right-153	TCGGCCCTGAATAAAGCCTCAGAGCATAAtttt
cuboid-right-154	ttttCCTGTTTAGGCTGCTCATtttt
cuboid-right-155	ttttAGCGGTCCACGCAGTGTTGTTCCAtttt
cuboid-right-156	ttttGCAACTAAAGGTCAATAAtttt
cuboid-right-157	ttttGGTCATTTTTTTAAATATtttt
cuboid-right-158	ATAAGATTAATTACCCTTGACCATAAATCAAAAtttt
cuboid-right-159	ttttCGAAAGACTTTGATAAGAtttt
cuboid-right-160	ttttATCAGGTCTGAGGAAGCCtttt
cuboid-right-161	ttttAGCGTCCAATACTGCGGAAACGAGAAGACTATTAAT
cuboid-right-162	ttttGCTTTTGCAAAATTTAGACTGGATGTAATACCAGATGG
cuboid-right-163	ttttCAGATACATAACAAATAGCGAGAGtttt
cuboid-right-164	ttttATTACAGGTAGAATTCAACTAATGGTAATACCAGATGG
cuboid-right-165	ttttGAATTACCTTATCGGAACAACATTtttt
cuboid-right-166	ttttATTACCCAAATCTTTAATCATTGTGTAATACCAGATGG
cuboid-right-167	ttttTGAGACGGGCAAAGAGTTGCAGCAGTAATACCAGATGG
cuboid-right-168	ttttCAACTTTGAAAGAGGAAGGGAACGCTCCATTAAA
cuboid-right-169	ATACAGATGATGACAAGAACCGGATATTCtttt
cuboid-right-170	ttttCCGCGACCTCGAACTGACtttt
cuboid-right-171	ttttAGAGGCAAATGTCGAAATtttt
cuboid-right-172	ttttTTCATGAGGTAAAACGAAtttt
cuboid-right-173	TTATATATTCTAGTTGCAATTTCTTAAACAGCTtttt
cuboid-right-174	ttttGGCTTGCAGAAAGACTTTtttt
cuboid-right-175	ttttTGATACCGAGGTCGCTGAtttt
cuboid-right-176	ttttCTCACTGCCCGCTCTTTTCACCAGtttt
cuboid-left-177	ttttCTTCTGTAAATCTGAAAACATTATATGGTCAACTG
cuboid-left-178	ttttATCTTTAGGAGCGAAGTATTAtttt

cuboid-left-179	ttttGACTTTACAAGAAACCAATCAtttt
cuboid-left-180	GCGTTTGCGGAGCAGCAGAGGAAGGTTATCTAAAATtttt
cuboid-left-181	ttttACCACCAGAAAAGGTAAAGTAtttt
cuboid-left-182	TGATATAATCCAGCAGACACCGCCTGCAACAGTGCCtttt
cuboid-left-183	ttttTGGATTATAAATTGAGAATCGtttt
cuboid-left-184	TGACAGAGATACATCGCCATTAAAAATACtttt
cuboid-left-185	ttttAACAGTACCCGACCGTGTGATtttt
cuboid-left-186	AAATGAAATGCGACCAGTAATAAAAGGGAtttt
cuboid-left-187	ttttAGGCGAATTCCAATCGCAAGAtttt
cuboid-left-188	ttttAGCGATAGCCAGATAGCCGAAtttt
cuboid-left-189	TAAGTTAGATTGAATCCTGTCGCTAGGAAATACCTACATTTTGAtttt
cuboid-left-190	ttttCAAAGTTACCAGTACCCAAAATATATGGTCAACTG
cuboid-left-191	ttttCAAAGAACGCGAACTGAACACtttt
cuboid-left-192	ttttAAATAAGGCGTTTAACGTCAATATATGGTCAACTG
cuboid-left-193	ttttCGCTCAATCGTCATCGCGCAGtttt
cuboid-left-194	TATTCGCTCATTTAATTATCAATATATGTGAGTGAATAACCTTGtttt
cuboid-left-195	ttttCCATATTTAACATACAATTTTtttt
cuboid-left-196	ttttATTCTGTCCAGAAGGCGTTTTTATATGGTCAACTG
cuboid-left-197	ttttATAATCGGCTGTTTCATCGTAtttt
cuboid-left-198	ttttGGAATCATTTTGAGGCAGGTCtttt
cuboid-left-199	ttttAGCGAACCTACCGGAACCAGAtttt
cuboid-left-200	ttttATCCTGAATGCCTTTAGCGTCtttt
cuboid-left-201	ttttAAATGAAAAACCGACTTGAGCtttt
cuboid-left-202	ttttCCTGAACAAGCCAAAGACAAAtttt
cuboid-left-203	ttttGAACTGGCAAGAATAGAAAGGtttt
cuboid-left-204	ttttAACAACTAAAGGAATTGTGTACCAGCAGTC
cuboid-left-205	ttttCATTCTGGCCAAATATACAGTTATATGGTCAACTG
cuboid-left-206	ttttAGGGCGACATTCCAGTACAAAtttt
cuboid-left-207	ttttCATTTGGGAATTCCTCAGAGCTATATGGTCAACTG
cuboid-left-208	ttttAGACTGTAGCGCTACCAGGCGtttt
cuboid-left-209	ttttGCCACCACCGGATATTATTCTTATATGGTCAACTG
cuboid-left-210	ttttAGACGATTGGCCAAGCGTCATtttt
cuboid-left-211	ttttACATGGCTTTTGATGATTCCAGTTTGATATTCAC
cuboid-left-212	ttttGAAACATGAAAGTATTCGGAACCACCGCCTCAGGAGGACC
cuboid-left-213	ttttGATAAGTGCCGTCGAGTGCTCAGGTTTTCACAAAATCCCC
cuboid-left-214	ttttCACCACCCTCATTTTCCCGCCACAGAGCCACAAG
cuboid-left-215	ttttCTACAACGCCTGTAGCTCGTCACAACCGATCACC
cuboid-left-216	ttttCGAACGAACCACCTGATTGTTtttt
cuboid-left-217	ttttTTCACGTTGAAAATCTTTCGAATAATAATTTTTATATGGTCAACTG
cuboid-left-218	ttttACGCTGAGAGCCAACAAAGAATATATGGTCAACTG

Table S8. Sequences of edge staples of DNA origami cuboid (valency number of 9). All sequences are written from 5' to 3'.

cuboid-right-135	ttttAACCGTCTATCATTGATTAGTAATAAGTGG
cuboid-right-136	ttttAAGTGTAAAGCCAATTGCGTTGCGGTAATACCAGATGG
cuboid-right-137	ttttCCTGTAATACTTTTGCCCAAAAAAGCTTGCCGTT
cuboid-right-138	GTCGAACGCAAGGATATAGGGAGAACATACGAGCCGGAAGCATAtttt
cuboid-right-139	ttttCCAGTGCCACATTATGACGTAATACCAGATGG
cuboid-right-140	ttttTCGGTGCGGAAACGACGGGTAATACCAGATGG
cuboid-right-141	ttttCGCTTCTGGGAAGGGCGAtttt
cuboid-right-142	ttttGTTTGGAACAAGCAAAGGGCGAAAGTAATACCAGATGG
cuboid-right-143	GGTGGATTGAGTGAGCGTAGCCAGCTTTCATCAtttt
cuboid-right-144	ttttGATAGGTCATTCCGGCACtttt
cuboid-right-145	ttttACATTAAATCCGTAATGGGTAATACCAGATGG
cuboid-right-146	ttttCATTAAATTTTTGTTAAATCTTCCTGAGTA
cuboid-right-147	ttttGAAGATTGTATATGTTAAAATTCGtttt
cuboid-right-148	ttttAGTCTGGAGCAACCCCAAAAACAGtttt
cuboid-right-149	ttttCGGAGAGGGTAGTCATTGCCTGAGtttt
cuboid-right-150	ttttGTAATGTGTAGGATAAATTAATGCtttt
cuboid-right-151	ttttAGCTAAATCGGTTGCAATGCCTGAtttt
cuboid-right-152	ttttTCAGTGAATAAGGCTTTAACAAACTATATTCGCA
cuboid-right-153	TCGGCCCTGAATAAAGCCTCAGAGCATAAtttt
cuboid-right-154	ttttCCTGTTTAGGCTGCTCATtttt
cuboid-right-155	ttttAGCGGTCCACGCAGTGTTGTTCCAGTAATACCAGATGG
cuboid-right-156	ttttGCAACTAAAGGTCAATAAtttt
cuboid-right-157	ttttGGTCATTTTTTTAAATATtttt
cuboid-right-158	ATAAGATTAATTACCCTTGACCATAAATCAAAAtttt
cuboid-right-159	ttttCGAAAGACTTTGATAAGAtttt
cuboid-right-160	ttttATCAGGTCTGAGGAAGCCtttt
cuboid-right-161	ttttAGCGTCCAATACTGCGGAAACGAGAAGACTATTAAT
cuboid-right-162	ttttGCTTTTGCAAAATTTAGACTGGATGTAATACCAGATGG
cuboid-right-163	ttttCAGATACATAACAAATAGCGAGAGGTAATACCAGATGG
cuboid-right-164	ttttATTACAGGTAGAATTCAACTAATGtttt
cuboid-right-165	ttttGAATTACCTTATCGGAACAACATTtttt
cuboid-right-166	ttttATTACCCAAATCTTTAATCATTGTGTAATACCAGATGG
cuboid-right-167	ttttTGAGACGGGCAAAGAGTTGCAGCAtttt
cuboid-right-168	ttttCAACTTTGAAAGAGGAAGGGAACGCTCCATTAAA
cuboid-right-169	ATACAGATGATGACAAGAACCGGATATTCtttt
cuboid-right-170	ttttCCGCGACCTCGAACTGACGTAATACCAGATGG
cuboid-right-171	ttttAGAGGCAAATGTCGAAATGTAATACCAGATGG
cuboid-right-172	ttttTTCATGAGGTAAAACGAAtttt
cuboid-right-173	TTATATATTCTAGTTGCAATTTCTTAAACAGCTtttt
cuboid-right-174	ttttGGCTTGCAGAAAGACTTTtttt
cuboid-right-175	ttttTGATACCGAGGTCGCTGAGTAATACCAGATGG
cuboid-right-176	ttttCTCACTGCCCGCTCTTTTCACCAGtttt
cuboid-left-177	ttttCTTCTGTAAATCTGAAAACATTATATGGTCAACTG
cuboid-left-178	ttttATCTTTAGGAGCGAAGTATTAtttt

cuboid-left-179	ttttGACTTTACAAGAAACCAATCAtttt
cuboid-left-180	GCGTTTGCGGAGCAGCAGAGGAAGGTTATCTAAAATTATATGGTCAACTG
cuboid-left-181	ttttACCACCAGAAAAGGTAAAGTAtttt
cuboid-left-182	TGATATAATCCAGCAGACACCGCCTGCAACAGTGCCTATATGGTCAACTG
cuboid-left-183	ttttTGGATTATAAATTGAGAATCGtttt
cuboid-left-184	TGACAGAGATACATCGCCATTAAAAATACtttt
cuboid-left-185	ttttAACAGTACCCGACCGTGTGATtttt
cuboid-left-186	AAATGAAATGCGACCAGTAATAAAAGGGAtttt
cuboid-left-187	ttttAGGCGAATTCCAATCGCAAGAtttt
cuboid-left-188	ttttAGCGATAGCCAGATAGCCGAAtttt
cuboid-left-189	TAAGTTAGATTGAATCCTGTCGCTAGGAAATACCTACATTTTGATATATGGTCAACTG
cuboid-left-190	ttttCAAAGTTACCAGTACCCAAAAtttt
cuboid-left-191	ttttCAAAGAACGCGAACTGAACACtttt
cuboid-left-192	ttttAAATAAGGCGTTTAACGTCAAtttt
cuboid-left-193	ttttCGCTCAATCGTCATCGCGCAGTATATGGTCAACTG
cuboid-left-194	TATTCGCTCATTTAATTATCAATATATGTGAGTGAATAACCTTGtttt
cuboid-left-195	ttttCCATATTTAACATACAATTTTtttt
cuboid-left-196	ttttATTCTGTCCAGAAGGCGTTTTtttt
cuboid-left-197	ttttATAATCGGCTGTTTCATCGTAtttt
cuboid-left-198	ttttGGAATCATTTTGAGGCAGGTCTATATGGTCAACTG
cuboid-left-199	ttttAGCGAACCTACCGGAACCAGATATATGGTCAACTG
cuboid-left-200	ttttATCCTGAATGCCTTTAGCGTCtttt
cuboid-left-201	ttttAAATGAAAAACCGACTTGAGCtttt
cuboid-left-202	ttttCCTGAACAAGCCAAAGACAAATATATGGTCAACTG
cuboid-left-203	ttttGAACTGGCAAGAATAGAAAGGtttt
cuboid-left-204	ttttAACAACTAAAGGAATTGTGTACCAGCAGTC
cuboid-left-205	ttttCATTCTGGCCAAATATACAGTtttt
cuboid-left-206	ttttAGGGCGACATTCCAGTACAAATATATGGTCAACTG
cuboid-left-207	ttttCATTTGGGAATTCCTCAGAGCtttt
cuboid-left-208	ttttAGACTGTAGCGCTACCAGGCGtttt
cuboid-left-209	ttttGCCACCACCGGATATTATTCTTATATGGTCAACTG
cuboid-left-210	ttttAGACGATTGGCCAAGCGTCATtttt
cuboid-left-211	ttttACATGGCTTTTGATGATTCCAGTTTGATATTCAC
cuboid-left-212	ttttGAAACATGAAAGTATTCGGAACCACCGCCTCAGGAGGACC
cuboid-left-213	ttttGATAAGTGCCGTCGAGTGCTCAGGTTTTCACAAAATCCCC
cuboid-left-214	ttttCACCACCCTCATTTTCCCGCCACAGAGCCACAAG
cuboid-left-215	ttttCTACAACGCCTGTAGCTCGTCACAACCGATCACC
cuboid-left-216	ttttCGAACGAACCACCTGATTGTTtttt
cuboid-left-217	ttttTTCACGTTGAAAATCTTTCGAATAATAATTTTTATATGGTCAACTG
cuboid-left-218	ttttACGCTGAGAGCCAACAAAGAATATATGGTCAACTG

Table S9. Sequences of edge staples of DNA origami cuboid (valency number of 12). All sequences are written from 5' to 3'.

cuboid-right-135	ttttAACCGTCTATCATTGATTAGTAATAAGTGG
cuboid-right-136	ttttAAGTGTAAAGCCAATTGCGTTGCGGTAATACCAGATGG
cuboid-right-137	ttttCCTGTAATACTTTTGCCCAAAAAAGCTTGCCGTT
cuboid-right-138	GTCGAACGCAAGGATATAGGGAGAACATACGAGCCGGAAGCATAGTAATACCAGATGG
cuboid-right-139	ttttCCAGTGCCACATTATGACGTAATACCAGATGG
cuboid-right-140	ttttTCGGTGCGGAAACGACGGGTAATACCAGATGG
cuboid-right-141	ttttCGCTTCTGGGAAGGGCGAtttt
cuboid-right-142	ttttGTTTGGAACAAGCAAAGGGCGAAAGTAATACCAGATGG
cuboid-right-143	GGTGGATTGAGTGAGCGTAGCCAGCTTTCATCAGTAATACCAGATGG
cuboid-right-144	ttttGATAGGTCATTCCGGCACGTAATACCAGATGG
cuboid-right-145	ttttACATTAAATCCGTAATGGGTAATACCAGATGG
cuboid-right-146	ttttCATTAAATTTTTGTTAAATCTTCCTGAGTA
cuboid-right-147	ttttGAAGATTGTATATGTTAAAATTCGGTAATACCAGATGG
cuboid-right-148	ttttAGTCTGGAGCAACCCCAAAAACAGGTAATACCAGATGG
cuboid-right-149	ttttCGGAGAGGGTAGTCATTGCCTGAGtttt
cuboid-right-150	ttttGTAATGTGTAGGATAAATTAATGCGTAATACCAGATGG
cuboid-right-151	ttttAGCTAAATCGGTTGCAATGCCTGAGTAATACCAGATGG
cuboid-right-152	ttttTCAGTGAATAAGGCTTTAACAAACTATATTCGCA
cuboid-right-153	TCGGCCCTGAATAAAGCCTCAGAGCATAAGTAATACCAGATGG
cuboid-right-154	ttttCCTGTTTAGGCTGCTCATGTAATACCAGATGG
cuboid-right-155	ttttAGCGGTCCACGCAGTGTTGTTCCAGTAATACCAGATGG
cuboid-right-156	ttttGCAACTAAAGGTCAATAAGTAATACCAGATGG
cuboid-right-157	ttttGGTCATTTTTTTAAATATtttt
cuboid-right-158	ATAAGATTAATTACCCTTGACCATAAATCAAAAGTAATACCAGATGG
cuboid-right-159	ttttCGAAAGACTTTGATAAGAGTAATACCAGATGG
cuboid-right-160	ttttATCAGGTCTGAGGAAGCCGTAATACCAGATGG
cuboid-right-161	ttttAGCGTCCAATACTGCGGAAACGAGAAGACTATTAAT
cuboid-right-162	ttttGCTTTTGCAAAATTTAGACTGGATGTAATACCAGATGG
cuboid-right-163	ttttCAGATACATAACAAATAGCGAGAGGTAATACCAGATGG
cuboid-right-164	ttttATTACAGGTAGAATTCAACTAATGtttt
cuboid-right-165	ttttGAATTACCTTATCGGAACAACATTGTAATACCAGATGG
cuboid-right-166	ttttATTACCCAAATCTTTAATCATTGTGTAATACCAGATGG
cuboid-right-167	ttttTGAGACGGGCAAAGAGTTGCAGCAtttt
cuboid-right-168	ttttCAACTTTGAAAGAGGAAGGGAACGCTCCATTAAA
cuboid-right-169	ATACAGATGATGACAAGAACCGGATATTCGTAATACCAGATGG
cuboid-right-170	ttttCCGCGACCTCGAACTGACGTAATACCAGATGG
cuboid-right-171	ttttAGAGGCAAATGTCGAAATGTAATACCAGATGG
cuboid-right-172	ttttTTCATGAGGTAAAACGAAtttt
cuboid-right-173	TTATATATTCTAGTTGCAATTTCTTAAACAGCTGTAATACCAGATGG
cuboid-right-174	ttttGGCTTGCAGAAAGACTTTGTAATACCAGATGG
cuboid-right-175	ttttTGATACCGAGGTCGCTGAGTAATACCAGATGG
cuboid-right-176	ttttCTCACTGCCCGCTCTTTTCACCAGGTAATACCAGATGG
cuboid-left-177	ttttCTTCTGTAAATCTGAAAACATTATATGGTCAACTG

cuboid-left-178	ttttATCTTTAGGAGCGAAGTATTATATATGGTCAACTG
cuboid-left-179	ttttGACTTTACAAGAAACCAATCATATATGGTCAACTG
cuboid-left-180	GCGTTTGCGGAGCAGCAGAGGAAGGTTATCTAAAATTATATGGTCAACTG
cuboid-left-181	ttttACCACCAGAAAAGGTAAAGTATATATGGTCAACTG
cuboid-left-182	TGATATAATCCAGCAGACACCGCCTGCAACAGTGCCTATATGGTCAACTG
cuboid-left-183	ttttTGGATTATAAATTGAGAATCGtttt
cuboid-left-184	TGACAGAGATACATCGCCATTAAAAATACtttt
cuboid-left-185	ttttAACAGTACCCGACCGTGTGATTATATGGTCAACTG
cuboid-left-186	AAATGAAATGCGACCAGTAATAAAAGGGATATATGGTCAACTG
cuboid-left-187	ttttAGGCGAATTCCAATCGCAAGATATATGGTCAACTG
cuboid-left-188	ttttAGCGATAGCCAGATAGCCGAATATATGGTCAACTG
cuboid-left-189	TAAGTTAGATTGAATCCTGTCGCTAGGAAATACCTACATTTTGATATATGG TCAACTG
cuboid-left-190	ttttCAAAGTTACCAGTACCCAAAATATATGGTCAACTG
cuboid-left-191	ttttCAAAGAACGCGAACTGAACACTATATGGTCAACTG
cuboid-left-192	ttttAAATAAGGCGTTTAACGTCAAtttt
cuboid-left-193	ttttCGCTCAATCGTCATCGCGCAGTATATGGTCAACTG
cuboid-left-194	TATTCGCTCATTTAATTATCAATATATGTGAGTGAATAACCTTGTATATGG TCAACTG
cuboid-left-195	ttttCCATATTTAACATACAATTTTTATATGGTCAACTG
cuboid-left-196	ttttATTCTGTCCAGAAGGCGTTTTTATATGGTCAACTG
cuboid-left-197	ttttATAATCGGCTGTTTCATCGTATATATGGTCAACTG
cuboid-left-198	ttttGGAATCATTTTGAGGCAGGTCTATATGGTCAACTG
cuboid-left-199	ttttAGCGAACCTACCGGAACCAGATATATGGTCAACTG
cuboid-left-200	ttttATCCTGAATGCCTTTAGCGTCtttt
cuboid-left-201	ttttAAATGAAAAACCGACTTGAGCTATATGGTCAACTG
cuboid-left-202	ttttCCTGAACAAGCCAAAGACAAATATATGGTCAACTG
cuboid-left-203	ttttGAACTGGCAAGAATAGAAAGGTATATGGTCAACTG
cuboid-left-204	ttttAACAACTAAAGGAATTGTGTACCAGCAGTC
cuboid-left-205	ttttCATTCTGGCCAAATATACAGTtttt
cuboid-left-206	ttttAGGGCGACATTCCAGTACAAATATATGGTCAACTG
cuboid-left-207	ttttCATTTGGGAATTCCTCAGAGCtttt
cuboid-left-208	ttttAGACTGTAGCGCTACCAGGCGTATATGGTCAACTG
cuboid-left-209	ttttGCCACCACCGGATATTATTCTTATATGGTCAACTG
cuboid-left-210	ttttAGACGATTGGCCAAGCGTCATTATATGGTCAACTG
cuboid-left-211	ttttACATGGCTTTTGATGATTCCAGTTTGATATTCAC
cuboid-left-212	ttttGAAACATGAAAGTATTCGGAACCACCGCCTCAGGAGGACC
cuboid-left-213	ttttGATAAGTGCCGTCGAGTGCTCAGGTTTTCACAAAATCCCC
cuboid-left-214	ttttCACCACCCTCATTTTCCCGCCACAGAGCCACAAG
cuboid-left-215	ttttCTACAACGCCTGTAGCTCGTCACAACCGATCACC
cuboid-left-216	ttttCGAACGAACCACCTGATTGTTTATATGGTCAACTG
cuboid-left-217	ttttTTCACGTTGAAAATCTTTCGAATAATAATTTTTATATGGTCAACTG
cuboid-left-218	ttttACGCTGAGAGCCAACAAAGAATATATGGTCAACTG

Table S10. Sequences of edge staples of DNA origami cuboid (valency number of 30).

All sequences are written from 5' to 3'.

A* (for 6-bt triplex)	GAAGAACAGTTGACCATATA
B* (for 6-bt triplex)	AAGAAGCCATCTGGTATTAC
A* (for 8-bt triplex)	GAAGAAGACAGTTGACCATATA
B* (for 8-bt triplex)	AGAAGAAGCCATCTGGTATTAC
A* (for 10-bt triplex)	GGGAAGAAGACAGTTGACCATATA
B* (for 10-bt triplex)	AGAAGAAGGGCCATCTGGTATTAC
A* (for 12-bt triplex)	AAGGGAAGAAGACAGTTGACCATATA
B* (for12-bt triplex)	AGAAGAAGGGAACCATCTGGTATTAC
A* (for 14-bt triplex)	AAGGAGAGAAGGAGCAGTTGACCATATA
B* (for 14-bt triplex)	GAGGAAGAGAGGAACCATCTGGTATTAC
strand C (6 nt)	TTCTTC
strand C (8 nt)	TCTTCTTC
strand C (10 nt)	TCTTCTTCCC
strand C (12 nt)	TCTTCTTCCCTT
strand C (14 nt)	CTCCTTCTCTCCTT

Table S11. Sequences of A* and B* for origami cuboids. All sequences are written from 5' to 3'.

A* (for input 1)	GGGAAGAAGACAGTTGACCATATA
B* (for input 1)	AGAAGAAGGGCCATCTGGTATTAC
A* (for input 2)	AGAGGAGGAACAGTTGACCATATA
B* (for input 2)	AAGGAGGAGACCATCTGGTATTAC
Input 1 (10 nt)	TCTTCTTCCC
Input 2 (10 nt)	TTCCTCCTCT

Table S12. Sequences of A* and B* for origami cuboids of selective signaling system. All sequences are written from 5' to 3'.

DBCO-DNA-FAM	DBCO-T_{22}-CATCCATCCTTATCAACT-FAM
A	DBCO-T_{20}-ACCGAGTTCAATAGGGGAGA
B	DBCO-T_{20}-ATGACTGTTTGAAGCACTCG
A10*	AGAGGAGGAATCTCCCCTATTGAACTCGGT
B10*	AAGGAGGAGACGAGTGCTTCAAACAGTCAT
A12*	AAGGGAAGAAGATCTCCCCTATTGAACTCGGT
B12*	AGAAGAAGGGAACGAGTGCTTCAAACAGTCAT
A16*	AAGGAAGGGAAGAAGATCTCCCCTATTGAACTCGGT
B16*	AGAAGAAGGGAAGGAACGAGTGCTTCAAACAGTCAT
A20*	AGAGAAGGAAGGGAAGAAGATCTCCCCTATTGAACTCGGT
B20*	AGAAGAAGGGAAGGAAGAGACGAGTGCTTCAAACAGTCAT
A24*	AAGGAGAGAAGGAAGGGAAGAAGATCTCCCCTATTGAACTCGGT
B24*	AGAAGAAGGGAAGGAAGAGAGGAACGAGTGCTTCAAACAGTCAT
strand C (10 nt)	TTCCTCCTCT
strand C (12 nt)	TCTTCTTCCCTT
strand C (16 nt)	TCTTCTTCCCTTCCTT
strand C (20 nt)	TCTTCTTCCCTTCCTTCTCT
strand C (24 nt)	TCTTCTTCCCTTCCTTCTCTCCTT

Table S13. Sequences of DNA triplex in bacterial surface engineering system. All sequences are written from 5' to 3'.

References.

[1] Kong, Y., Du, Q., Li, J. and Xing, H. (2022) Engineering bacterial surface interactions using DNA as a programmable material. *Chem. Commun.*, **58**, 3086-3100.
